# Oxaliplatin disrupts nucleolar function through biophysical disintegration

**DOI:** 10.1016/j.celrep.2022.111629

**Published:** 2022-11-08

**Authors:** H. Broder Schmidt, Zane A. Jaafar, B. Erik Wulff, Jason J. Rodencal, Kibeom Hong, Mohammad O. Aziz-Zanjani, Peter K. Jackson, Manuel D. Leonetti, Scott J. Dixon, Rajat Rohatgi, Onn Brandman

**Affiliations:** 1 Department of Biochemistry, Stanford University School of Medicine, Stanford, CA, USA; 2 Department of Medicine, Stanford University School of Medicine, Stanford, CA, USA; 3 Stanford Cancer Institute, Stanford University School of Medicine, Stanford, CA, USA; 4 Baxter Laboratory for Stem Cell Biology, Stanford University School of Medicine, Stanford, CA, USA; 5 Department of Biology, Stanford University, Stanford, CA, USA; 6 Chan Zuckerberg Biohub, San Francisco, CA, USA; 7 Lead contact

## Abstract

Platinum (Pt) compounds such as oxaliplatin are among the most commonly prescribed anti-cancer drugs. Despite their considerable clinical impact, the molecular basis of platinum cytotoxicity and cancer specificity remain unclear. Here we show that oxaliplatin, a backbone for the treatment of colorectal cancer, causes liquid-liquid demixing of nucleoli at clinically relevant concentrations. Our data suggest that this biophysical defect leads to cell-cycle arrest, shutdown of Pol I-mediated transcription, and ultimately cell death. We propose that instead of targeting a single molecule, oxaliplatin preferentially partitions into nucleoli, where it modifies nucleolar RNA and proteins. This mechanism provides a general approach for drugging the increasing number of cellular processes linked to biomolecular condensates.

## In brief

Schmidt et al. report on the mechanism of the anti-cancer drug oxaliplatin. They find that oxaliplatin affects phase separation, disintegrates nucleoli, and indirectly inhibits rRNA synthesis. This suggests that targeting the nucleolus with drugs modulating its biophysical properties is a viable anti-cancer strategy.

## Graphical abstract

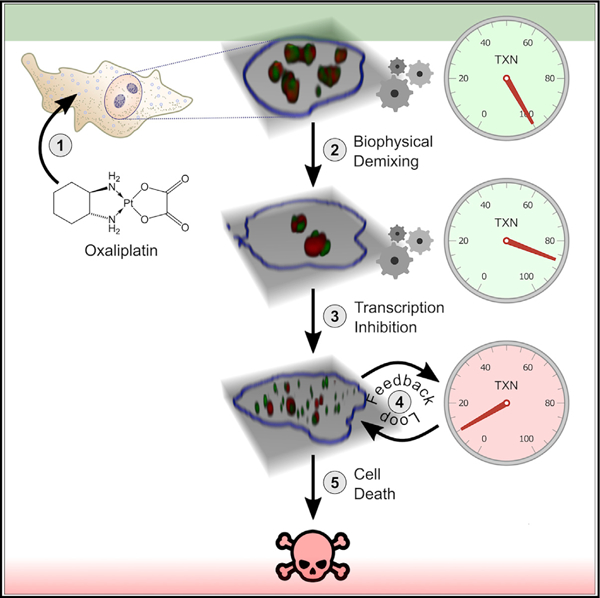

## INTRODUCTION

Platinum (Pt) compounds (Figure S1) were discovered serendipitously more than fifty years ago when the electrolysis products of platinum electrodes were shown to inhibit the growth of *E. coli*.^1^ Cisplatin, the first platinum compound to be developed as a result of these pioneering studies, soon became the cornerstone of treatment for ovarian, lung, head and neck, bladder and germ cell cancers.^2^ Significant toxicities, including kidney damage, nausea and vomiting, hearing changes, and peripheral neuropathy, led to the development of additional platinum analogs, two of which (carboplatin and oxaliplatin) are used clinically.^1^ Oxaliplatin is particularly effective in colorectal cancer (CRC), a disease in which cisplatin and carboplatin have no meaningful activity.^2^ Yet the mechanism of action (MOA) by which oxaliplatin kills cells, in particular colorectal cancer cells, has remained elusive. Understanding the MOA of oxaliplatin and other platinum compounds promises to unlock new principles in how to selectively target specific cancers.

Although the highly reactive platinum warhead common to Pt drugs can form adducts with all classes of cellular macromolecules (DNA, RNA, and proteins),^3^ the dominant view is that their cytotoxicity is caused by the formation of intrastrand adducts with purine bases in DNA, ultimately leading to failed DNA-damage responses.^4,5^ This model is based partly on the correlation between cytotoxicity and the abundance of specific G-G and A-G DNA-Pt adducts.^6^ However, recent biochemical and drug profiling studies have suggested a fundamentally different MOA unique to oxaliplatin over other Pt drugs: ribosome biogenesis stress.^7–9^

In this work, we set out to understand the molecular basis for how oxaliplatin inhibits ribosome biogenesis, motivated by the possibility that this mechanism may help improve the therapeutic profile of oxaliplatin and, more important, identify a new cellular pathway that could be targeted for cancer therapy. Clues come from the recent observations that oxaliplatin accumulates in nucleoli, inhibits rRNA transcription and alters nucleolar morphology.^7,10–12^ Nucleoli are phase-separated, multi-layered protein and RNA condensates that are the cellular factories for rRNA transcription, rRNA processing and ribosome assembly.^13^ In mammals, nucleoli contain three subphases: the fibrillar center (FC) where rRNA is transcribed, the dense fibrillar component (DFC) where rRNA is processed, and the granular component (GC) where ribosomal subunits are assembled.^14^ Notably, each nucleolar subphase is not only enriched for a specific set of protein and RNA species but also has distinct biophysical properties such as surface tension and viscoelasticity.^13^ The different biophysical properties of the nucleolar subphases are crucial for the sequential assembly of ribosomes by forming a dynamic ‘‘assembly line.’’^15–17^

Here, we show that oxaliplatin causes the biophysical disintegration of both the overall multi-phase organization of nucleoli and individual nucleolar sublayers at clinically relevant concentrations, and suggest that oxaliplatin indirectly disrupts rRNA transcription by collapsing the nucleolar assembly line. Because rRNA itself is an essential biophysical scaffold, we propose that oxaliplatin triggers a positive feedback loop: demixing impairs rRNA transcription, which begets further biophysical changes in nucleolar properties. The ultimate consequences are collapse of nucleolar function and defects in ribosome biogenesis. Our findings suggest that targeting the biophysical properties of nucleoli can perturb their function, opening chemotherapeutic avenues for translation-addicted cancers such as CRCs.^8,18^

## RESULTS

### Approach to uncover the oxaliplatin MOA

In this work, we sought to uncover how oxaliplatin causes ribosome biogenesis stress. The findings that oxaliplatin inhibits rRNA synthesis and alters nucleolar morphology can be explained by two models (Figure S1A). In the first model, oxaliplatin acts like the RNA polymerase inhibitor actinomycin D and blocks rRNA transcription.^19^ This in turn drives alterations in nuclear morphology, as rRNA is a determinant of nucleolar form and material properties.^16,20–22^ In the second model, oxaliplatin affects nucleoli independently of transcription, and this defect then secondarily leads to impaired transcription. As the biophysical form and rRNA biogenesis function of nucleoli are inextricably linked,^16,21^ oxaliplatin could trigger this cascade by interfering with the phase separation behavior of nucleoli.

To distinguish between these models, we performed *in vivo* and *in vitro* experiments to measure the effects of oxaliplatin on nucleolar biophysics and transcriptional activity. Oxaliplatin was compared with actinomycin D and cisplatin, a platinum drug not known to have effects on ribosome biogenesis^8^ (Figure S1B). As experimental systems, we chose CRC (HCT116) and sarcoma (U2OS) cells. We generated HCT116 and U20S cell lines in which the nucleolar scaffold components FBL (DFC marker) and NPM1 (GC marker) were epitope-tagged at their endogenous gene loci with fluorescent proteins (sfGFP and mTagRFP, respectively). Because of their CRC origin, we predicted and confirmed that HCT116 cells are more sensitive to oxaliplatin than U2OS (Figure S2), allowing insights into the cell type selectivity of oxaliplatin.

### Oxaliplatin increases nucleolar surface tension

**Figure 1.**
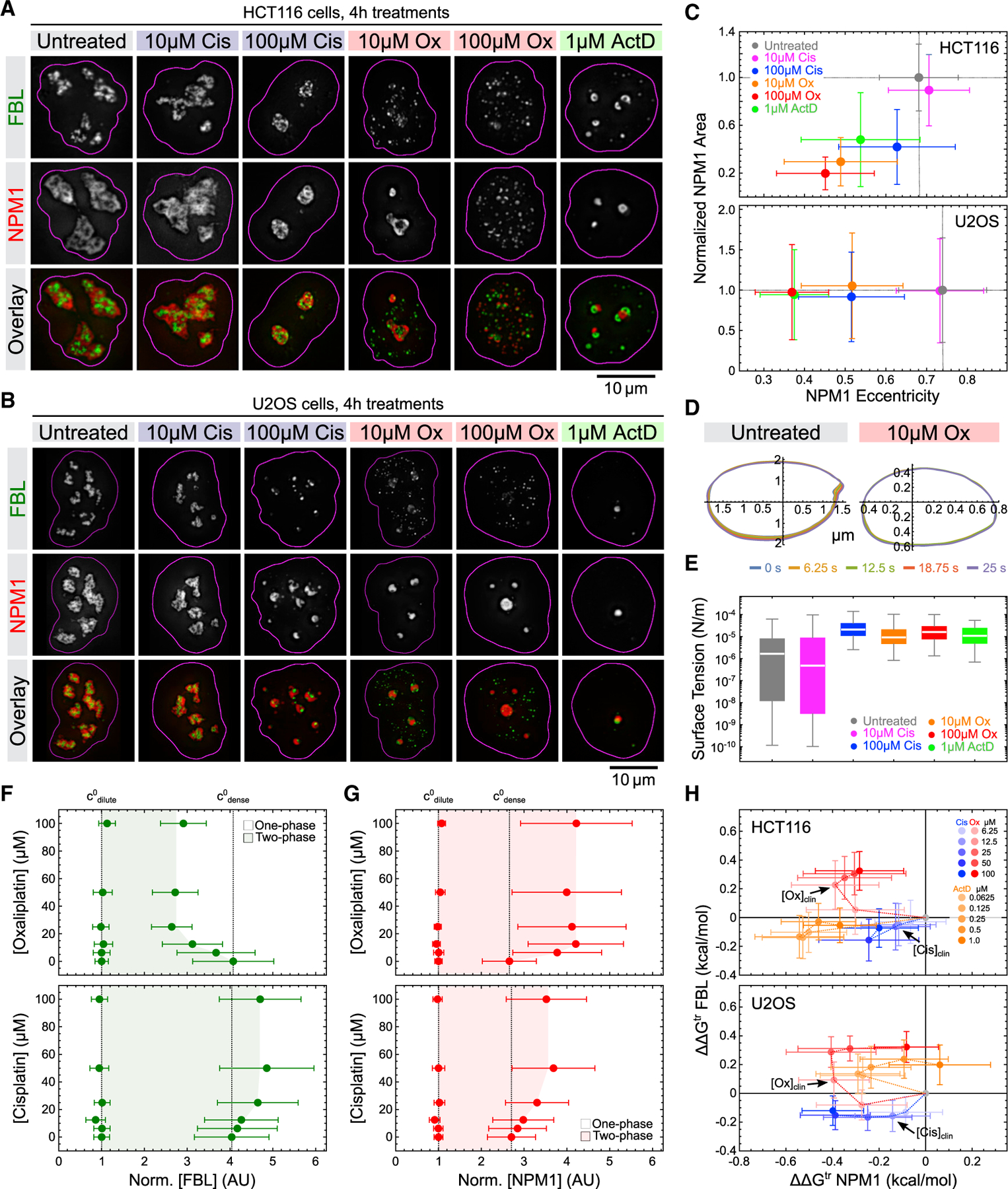
Oxaliplatin differentially modulates the driving forces of FBL and NPM1 phase separation (A and B) Representative deconvoluted epifluorescence images of HCT116 (A) and U2OS (B) cells expressing FBL-GFP (green) and NPM1-RFP (red) from their endogenous loci after 4 h treatments with the indicated amount of drugs. (C) Quantification of the changes in nucleolar eccentricity (x axis) and nucleolar area (y axis; normalized to untreated) in HCT116 and U2OS cells treated with the indicated amounts of drugs, measured using the nucleolar marker NPM1. Plot markers represent median values and error bars median deviation. At least 582 nucleoli per condition and cell line were quantified (see Data S1 for details). (D) Representative plots showing nucleolar surface contour fluctuations over time in U2OS cells after treatment with 0 or 10 uM oxaliplatin (Ox) for 4 h. Color of traces represent different time points as specified in the legend. (E) Quantification of the changes in NPM1 surface tension in response to drug treatments. Horizontal lines depict median values, boxes the 25th to 75th percentiles, and error bars the 5th to 95th percentiles. n = 136 (untreated), 200 (10 μM cisplatin [Cis]), 217 (100 μM Cis), 178 (10 μM Ox), 167 (100 μM Ox), and 40 (1 μM ActD). See Data S1 for statistics. (F and G) Phase diagrams showing changes in the dilute and dense-phase concentrations (in arbitrary units [AU]) of FBL-GFP (F) and NPM1-RFP (G) in HCT116 cells treated with increasing concentrations of oxaliplatin and cisplatin. Shaded areas indicate approximate two-phase regimes in which FBL-GFP and NPM1-RFP exist in condensates. Plot markers represent median values and error bars median deviation. At least 268 nucleoli per condition and cell line were quantified (see Data S1 for details). (H) Changes in FBL and NPM1 transfer energies (∆∆G^tr^) in HCT116 and U2OS cells treated with the indicated amounts of cisplatin (blue shades), oxaliplatin (red shades), and actinomycin D (orange shades) for 4 h (relative to untreated HCT116 and U2OS cells). The arrows indicate clinical concentrations of cisplatin and oxaliplatin. Plot markers represent median values and error bars median deviation. At least 268 nucleoli per condition and cell line were quantified (see Data S1 for details).

To validate and benchmark our experimental systems, we first determined the effect of oxaliplatin, cisplatin, and actinomycin D on nucleolar morphology. Following treatment of both HCT116 and U2OS cells with clinically relevant concentrations of oxaliplatin (~10 μM^7^), we observed three major changes in nucleolar morphology (Figures 1A and 1B). First, FBL was no longer nested within the NPM1 phase as in untreated cells. Second, FBL instead localized to numerous small foci throughout the nucleus, most of which no longer showed any association with NPM1. Third, both the FBL and NPM1 phases became more spherical. In HCT116 cells, we also observed the fragmentation of NPM1 into multiple small foci at high oxaliplatin doses (Figure 1C). Notably, these changes were qualitatively different to the morphological changes induced by cisplatin and actinomycin D, neither of which caused the widespread fragmentation of FBL. Moreover, cisplatin disrupted nucleolar morphology only at a concentration of 100 μM, which is well above clinically relevant concentrations (~10 μM^7^). Actinomycin D caused a distinct morphological change, including the rapid formation of ‘‘nucleolar caps,’’ structures that have been associated with transcriptional arrest and cellular stress^23,24^ (Figures 1A–1C). Quantitative insights into the biophysical driving forces underlying these morphological phenotypes may help us distinguish between the different models for the oxaliplatin MOA.

Surface tension has been previously identified as a key determinant of nucleolar ultrastructure.^25^ By measuring nucleolar surface fluctuations over time,^26^ we estimated that the surface tension at the NPM1-nucleoplasm interface increased by an order of magnitude in U2OS cells treated with 10 and 100 μM oxaliplatin, 100 μM cisplatin, and 1 μM actinomycin D (Figures 1D and 1E). In contrast to untreated cells, the increased sphericity and surface tension of NPM1 in cells treated with oxaliplatin, actinomycin D, and high doses of cisplatin indicate a significant change in their fluid properties from complex, non-Newtonian toward simple liquid behavior.^22^ These observations demonstrate that oxaliplatin, unlike cisplatin, affects key driving forces that control the stereotypic multi-phase morphology of nucleoli at clinically relevant concentrations.

### Oxaliplatin alters nucleolar phase separation

We next sought to determine how oxaliplatin affects the phase separation of the major DFC and GC scaffolds FBL and NPM1, respectively. This can be quantitatively described by two-dimensional phase diagrams, where one axis typically is concentration and the other axis a biologically relevant quantity that tunes the protein-protein and protein-solvent interactions underlying the phase transition (i.e., the Flory interaction parameter), such as salt or temperature.^27,28^ If oxaliplatin were indeed to alter the phase separation of FBL and NPM1, it likewise should tune the Flory parameter and shape the two-phase regime in which FBL and NPM1 phases exist.

To test this, we treated HCT116 cells with varying concentrations of oxaliplatin and measured the FBL and NPM1 fluorescence intensities (as a proxy for concentration) in the nucleoplasm (dilute phase) and nucleoli (dense phase) (Figures 1F, 1G, and S3). Unexpectedly, we observed that FBL and NPM1 behaved in diametrically opposite ways: whereas the FBL dense-phase concentration declined with increasing oxaliplatin concentrations (Figure 1F), the NPM1 dense-phase concentration increased (Figure 1G). As the dilute phase concentrations of both proteins were unaffected, oxaliplatin thus markedly limited the two-phase regime for FBL and expanded it for NPM1. In contrast, cisplatin caused a slight expansion of the two-phase regime for both FBL and NPM1 (Figures 1F and 1G). These data suggest that oxaliplatin oppositely tunes the strength of the interactions driving FBL (down) and NPM1 (up) phase separation.

To quantitatively describe the changes in phase separation driving force, we calculated the transfer energies ∆G^tr^ of FBL and NPM1 from the nucleoplasm (dilute phase) into nucleoli (dense phase), as previously described.^16^ ∆G^tr^ is a function of the ‘‘width’’ of the two-phase regime (i.e., the ratio of the dense-phase concentration over the light-phase concentration; Figure S3). Using ∆G^tr^ in untreated cells as a reference, we then determined the changes in ∆G^tr^ induced by a range of concentrations of oxaliplatin, cisplatin, and actinomycin D in both HCT116 and U2OS cells. This allowed us to construct thermodynamic maps for the direct comparison of drug effects on FBL and NPM1 phase separation, revealing three insights (Figure 1H). First, oxaliplatin oppositely affects ∆∆G^tr^ of FBL and NPM1 in both HCT116 and U2OS cells, causing it to be positive for FBL and negative for NPM1. This corresponds to a reduced thermodynamic driving force for FBL phase separation and an increased one for NPM1, in agreement with the observed changes in nucleolar morphology. Second, with increasing doses of oxaliplatin, ∆∆G^tr^ for NPM1 became less negative, demonstrating a reversal in the driving force for NPM1 phase separation. HCT116 cells were more sensitive for this reversal, as it occurred at oxaliplatin doses greater than 12.5 μM as opposed to 25 μM for U2OS cells. Notably, this mimics the greater overall sensitivity of HCT116 cells to oxaliplatin compared with U2OS cells (Figure S2), suggesting that altered nucleolar phase separation drives the effects of oxaliplatin on cell viability. Third, cisplatin showed a distinct thermodynamic profile compared with oxaliplatin, as it reduced ∆∆G^tr^ of both FBL and NPM1. Notably, actinomycin D had the same effect as cisplatin in HCT116 cells but not in U2OS cells, in which it rather caused a reduction in FBL and an increase in NPM1 phase separation, similar to oxaliplatin.

Together, our data show that oxaliplatin robustly disrupts the multi-phase organization of nucleoli in two different cell types by affecting the phase separation thermodynamics of key nucleolar scaffold proteins. In contrast, cisplatin stabilizes nucleolar ultrastructure at clinically relevant concentrations. Thus, even though both drugs carry the same Pt warhead, their effects on nucleolar morphology are strikingly divergent at clinical concentrations.

### Oxaliplatin disintegrates the granular component subphase

**Figure 2.**
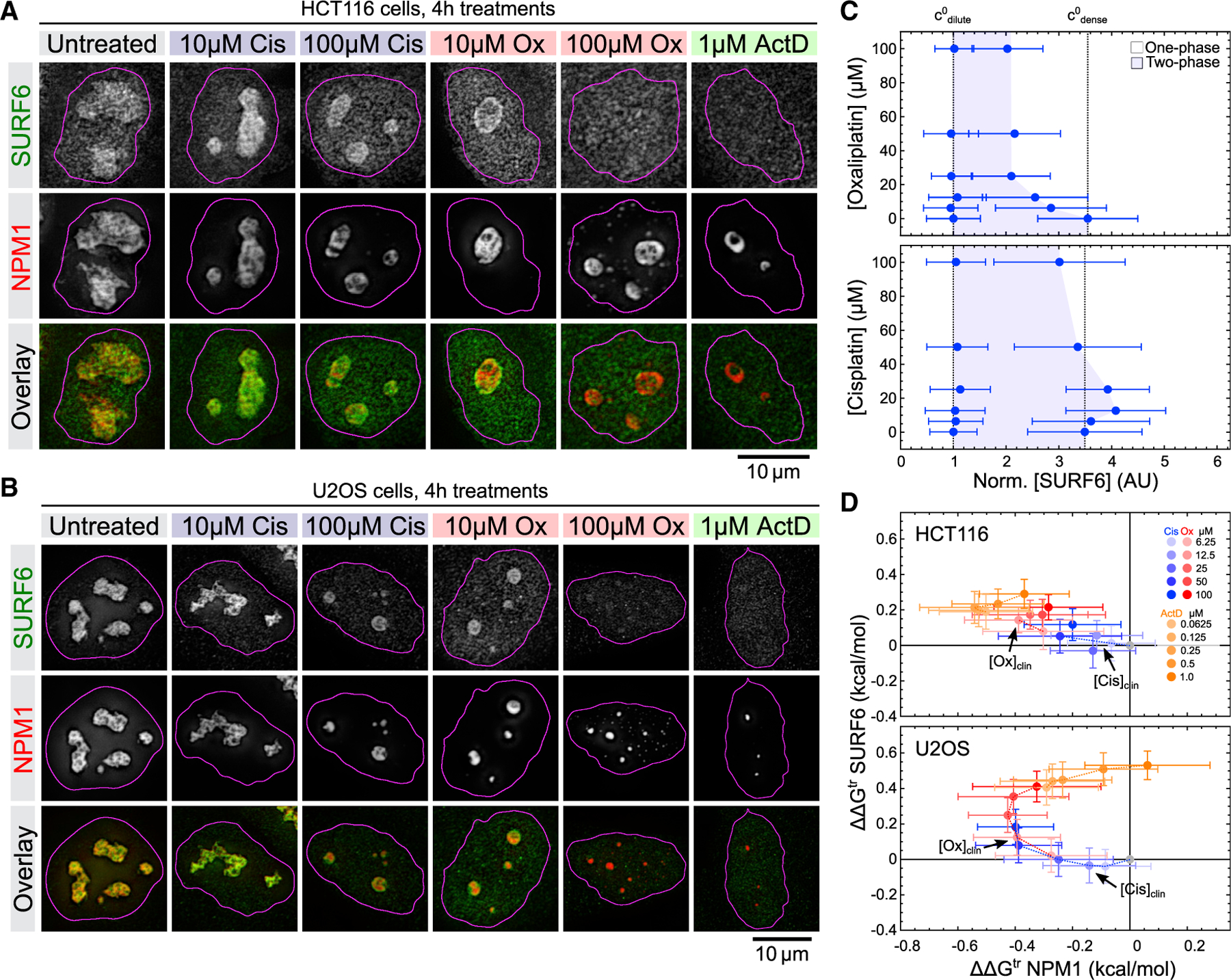
Oxaliplatin demixes the granular component phase of nucleoli (A and B) Representative deconvoluted epifluorescence images of immunostained SURF6 (green) in HCT116 (A) and U2OS (B) cells expressing NPM1-RFP (red) from its endogenous locus after 4 h treatments with the indicated amount of drugs. (C) Phase diagrams showing changes in the dilute and dense-phase concentrations (in AU) of SURF6 in HCT116 cells treated with increasing concentrations of oxaliplatin and cisplatin. Shaded areas indicate approximate two-phase regimes in which SURF6 exists in condensates. Plot markers represent median values and error bars median deviation. At least 268 nucleoli per condition and cell line were quantified (see Data S1 for details). (D) Changes in SURF6 and NPM1 transfer energies (∆∆G^tr^) in HCT116 and U2OS cells treated with the indicated amounts of cisplatin (blue shades), oxaliplatin (red shades), and actinomycin D (orange shades) for 4 h (relative to untreated HCT116 and U2OS cells). The arrows indicate clinical concentrations of cisplatin and oxaliplatin. Plot markers represent median values and error bars median deviation. At least 268 nucleoli per condition and cell line were quantified (see Data S1 for details).

Although FBL and NPM1 are established markers of the DFC and GC, they are not the sole scaffold components of their respective nucleolar layers. This poses the question how oxaliplatin affects individual nucleolar subphases. To test this, we focused on the well-studied interaction between NPM1 and SURF6, which form the scaffold of the GC.^15,20,29^ Although NPM1 and SURF6 co-localize in both untreated HCT116 and U2OS cells, oxaliplatin treatment caused the redistribution of SURF6 to the NPM1-nucleoplasm interface (Figures 2A and 2B), again reflecting changes in surface tension in response to oxaliplatin treatment. At oxaliplatin concentrations above 20 μM, SURF6 is nearly completely lost from nucleoli (Figures 2A–2C). We observed a similar phenotype for actinomycin D treatment, whereas cisplatin showed only mild effects on nucleoli at elevated concentrations (Figures 2A–2C).

To gain quantitative insights into the oxaliplatin-induced changes in the NPM1-SURF6 interaction and phase separation, we calculated ∆∆G^tr^ for NPM1 and SURF6. In both HCT116 and U2OS cells, increasing concentrations of oxaliplatin caused ∆∆G^tr^ (SURF6) to become more positive and ∆G^tr^ (SURF6) more negative (Figure 2D). This is consistent with the destabilization of the NPM1-SURF6 interaction and demixing of the GC phase due to increased NPM1 and decreased SURF6 phase separation. In contrast, cisplatin only affected the ∆∆G^tr^ of SURF6 well above clinical concentrations in both HCT116 and U2OS cells (Figure 2D). Actinomycin D acted similar to oxaliplatin in HCT116 cells, whereas in U2OS it caused the ∆∆G^tr^ of both SURF6 and NPM1 to become positive with increasing doses, indicative of nucleolar dissolution (Figure 2D). We conclude that oxaliplatin and actinomycin D (but not cisplatin) cause the collapse of the core GC scaffold, leading to a liquid-liquid demixing of the GC phase at clinical concentrations, which is consistent with MOAs that target nucleoli.

### Oxaliplatin disrupts nucleolar dynamics during the cell cycle

**Figure 3.**
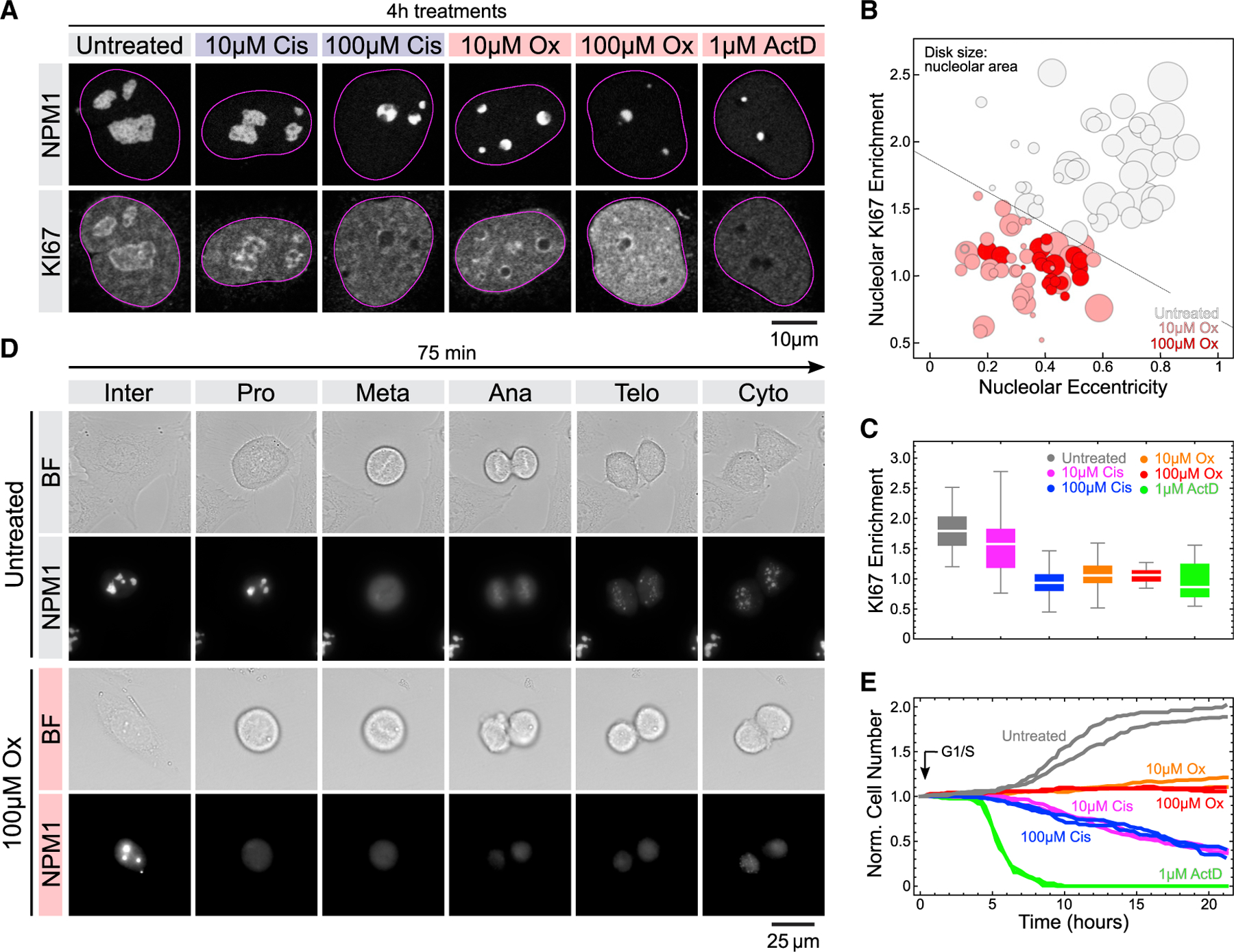
Oxaliplatin treatment disrupts the nucleolar rim compartment and causes cell cycle defects (A) Representative confocal images of NPM1-RFP (red) expressing U2OS cells immunostained for the nucleolar rim and cell proliferation marker KI67 (green) after 4 h treatments with the indicated amount of drugs. (B) Quantification of nucleolar KI67 enrichment (y axis), nucleolar eccentricity (x axis) and nucleolar area (plot marker size) in oxaliplatin-treated cells. At least 20 nucleoli per condition were analyzed. See Data S1 for statistics. (C) Quantification of nucleolar KI67 enrichment in oxaliplatin-, cisplatin- and actinomycin D-treated cells. Horizontal lines depict median values, boxes the 25th to 75th percentiles, and error bars the 5th to 95th percentiles. At least 15 nucleoli per condition were analyzed. See Data S1 for statistics. (D) Live cell imaging of nucleolar dynamics during mitosis in control and oxaliplatin-treated U2OS cells expressing NPM1-RFP from its endogenous locus. (E) Quantification of cell number during live cell imaging of control and drug-treated cells for 24 h. At least 92 cells per replicate were tracked over time (see Data S1 for details).

In addition to the scaffold components that drive phase separation and define material properties, biomolecular condensates such as nucleoli also contain various client molecules that interact with the scaffolds.^30^ To test how oxaliplatin affects client recruitment to nucleoli, we looked at the cell proliferation and cancer prognosis marker KI67.^31^ In untreated cells, KI67 localized to the nucleolar rim (Figure 3A), as previously reported.^32^ Given the surfactant-like properties of KI67,^33^ we expected that its association with nucleoli would be highly sensitive to the changes in nucleolar surface tension induced by oxaliplatin. Indeed, we found that low doses of oxaliplatin caused the loss of KI67 from the nucleolar rim and its dispersion into the nucleoplasm (Figures 3A–3C), providing further evidence that platination alters both biochemical interactions and biophysical forces within nucleoli.

During mitosis, nucleoli are disassembled and KI67 is localized to mitotic chromosomes, where it plays a key role in chromosome dispersal and nuclear reassembly.^33,34^ Notably, KI67 chromosome association is also a prerequisite for NPM1 recruitment and the reformation of nucleoli.^35^ This prompted us to investigate the effect of oxaliplatin on nucleoli during mitosis in synchronized U2OS cells with live cell imaging (Figure S4). Using endogenously tagged NPM1 as a marker, we found that the nucleoli of both untreated and oxaliplatin-treated cells disassemble following nuclear envelope breakdown at the end of prophase (Figure 3D). However, in oxaliplatin-treated cells, NPM1 failed to localize to mitotic chromosomes during anaphase and nucleoli failed to reform during telophase (Figure 3D). Notably, most oxaliplatin-treated cells failed to enter the cell cycle (Figure 3E), in agreement with previous reports.^36^ We could not observe or assess nucleolar reformation in cisplatin- and actinomycin D-treated cells because of immediate toxicity, (Figure 3E). These data show that the oxaliplatin-induced defects in nucleoli persist throughout the cell cycle and prevent the reformation of nucleoli after mitosis.

### Oxaliplatin indirectly interferes with ribosomal RNA transcription

Having established that oxaliplatin causes nucleolar demixing, we next sought to test how it affects ribosome biogenesis, which is the core function of nucleoli.^14^ This process includes three main steps: transcription of the 45S pre-ribosomal RNA (rRNA); processing of the pre-rRNA into mature 28S, 18S, and 5.8S rRNAs; and assembly of the processed rRNAs and ribosomal proteins into ribosomal subunits.^37^

**Figure 4.**
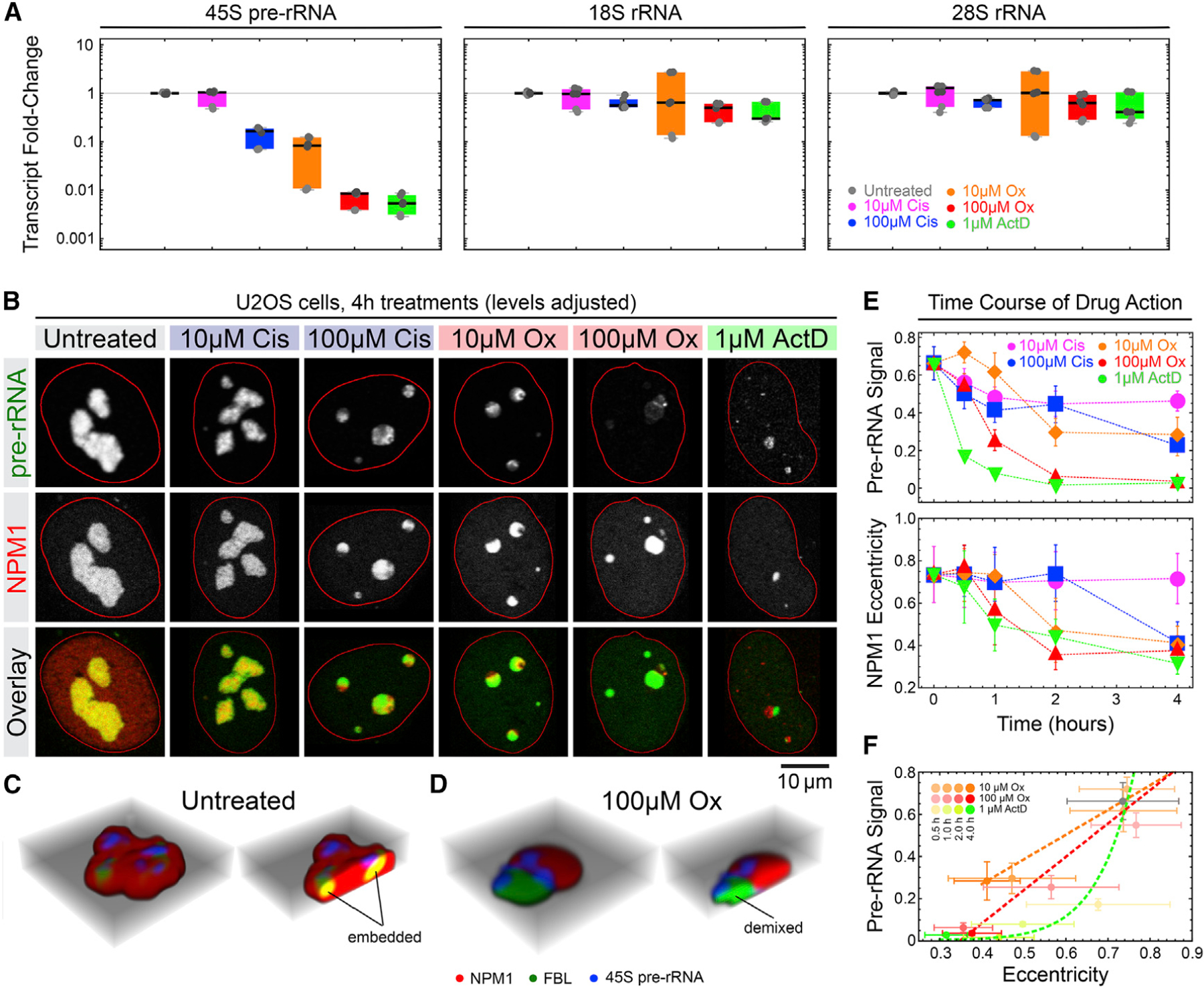
Oxaliplatin interferes with the transcription and sub-nucleolar localization of rRNA (A) Transcript levels of pre- and mature rRNA levels in U2OS knockin cells after treatment with the indicated amounts of drugs for 4 h, measured by qRT-PCR. Black horizontal lines indicate median values, boxes the the 25th to 75th percentiles, and error bars the 5th to 95th percentiles. Gray shading of data points represents technical replicates are shaded in gray according to. (B) Representative confocal images of RNA-FISH stainings against the 45S pre-rRNA (green) in U2OS knockin cells expressing NPM1-RFP (red) from its endogenous locus. The cells were treated with the indicated amounts of drugs for 4 h prior to staining. Image levels were individually adjusted to highlight morphological phenotypes. (C and D) Multi-channel three-dimensional (3D) reconstructions of a representative nucleoli in cells left untreated (C) or treated with 10 μM oxaliplatin for 4 h (D). (E and F) Quantification of the decline in 45S pre-rRNA signal and NPM1 eccentricity over time in U2OS knockin cells treated with the indicated amounts of drugs over time. Plot markers represent median values and error bars median deviation. At least 106 nucleoli per condition and time point were quantified in raw images (see Data S1 for details).

We focused on the first step and measured the total cellular levels of the 45S pre-rRNA, 18S rRNA, and 28S rRNA in U2OS cells after four hour drug treatments using quantitative reverse transcription PCR (qPCR). In cells treated with 10 μM oxaliplatin, we observed a greater than 10-fold reduction in 45S pre-rRNA levels, while the levels of the mature 18S and 28S rRNAs remained unaffected (Figure 4A). In contrast, clinical doses of cisplatin (10 μM) neither affected the pre-rRNA nor the mature rRNAs. Treatment with 100 μM oxaliplatin resulted in a ~100-fold reduction of the 45S pre-rRNA levels and a small but significant drop in 18S and 28S rRNA levels, similar to the RNA polymerase inhibitor actinomycin D (Figure 4A; Data S1). Consistent with other reports,^7,9^ we conclude that oxaliplatin interferes with rRNA transcription at clinically relevant doses, which ultimately leads to depletion of cellular ribosome levels.

To elucidate how the effects of oxaliplatin on rRNA transcription relates to its effects on nucleolar biophysics, we performed fluorescence *in situ* hybridization (FISH) against the 45S pre-rRNA in U2OS cells with endogenously tagged NPM1 after varying durations of drug treatment. To capture newly synthesized rRNA, we designed our FISH probes to target the short-lived 5′ETS region of the 45S pre-rRNA.^37^ As expected, we detected strong FISH signals that co-localized with NPM1 in untreated cells (Figure 4B). In agreement with our qPCR results, we observed a clear reduction in nucleolar FISH signal in cells treated with 10 μM oxaliplatin or 100 μM cisplatin and hardly any signal in cells treated with 100 μM oxaliplatin or 1 μM actinomycin D (Figure 4B). Our FISH data moreover revealed a dramatic delocalization of the 45S pre-rRNA concomitant with the demixing of the FBL and NPM1 subphases. Instead of being embedded within the GC together with FBL as in untreated cells, any remaining 45S pre-rRNA molecules were enriched at the interface of two nearly completely separated FBL and NPM1 phases in oxaliplatin-treated cells (Figures 4C and 4D). Remarkably, time course analysis revealed that oxaliplatin decreased 45S pre-rRNA levels and demixed nucleoli at a similar rate, whereas the RNA polymerase inhibitor actinomycin D first affected rRNA levels and only then altered nucleolar morphology (Figures 4E and 4F). This result is consistent with past studies demonstrating that, even at low doses, actinomycin D causes loss in transcription before nucleolar morphology changes.^38^ Together, this suggests that oxaliplatin acts through a mechanism distinct from actinomycin D, a direct inhibitor of Pol I, and thus blocks rRNA transcription through an indirect mechanism.

### Oxaliplatin-mediated nucleolar demxing and rRNA transcriptional shutdown are temporally correlated

**Figure 5.**
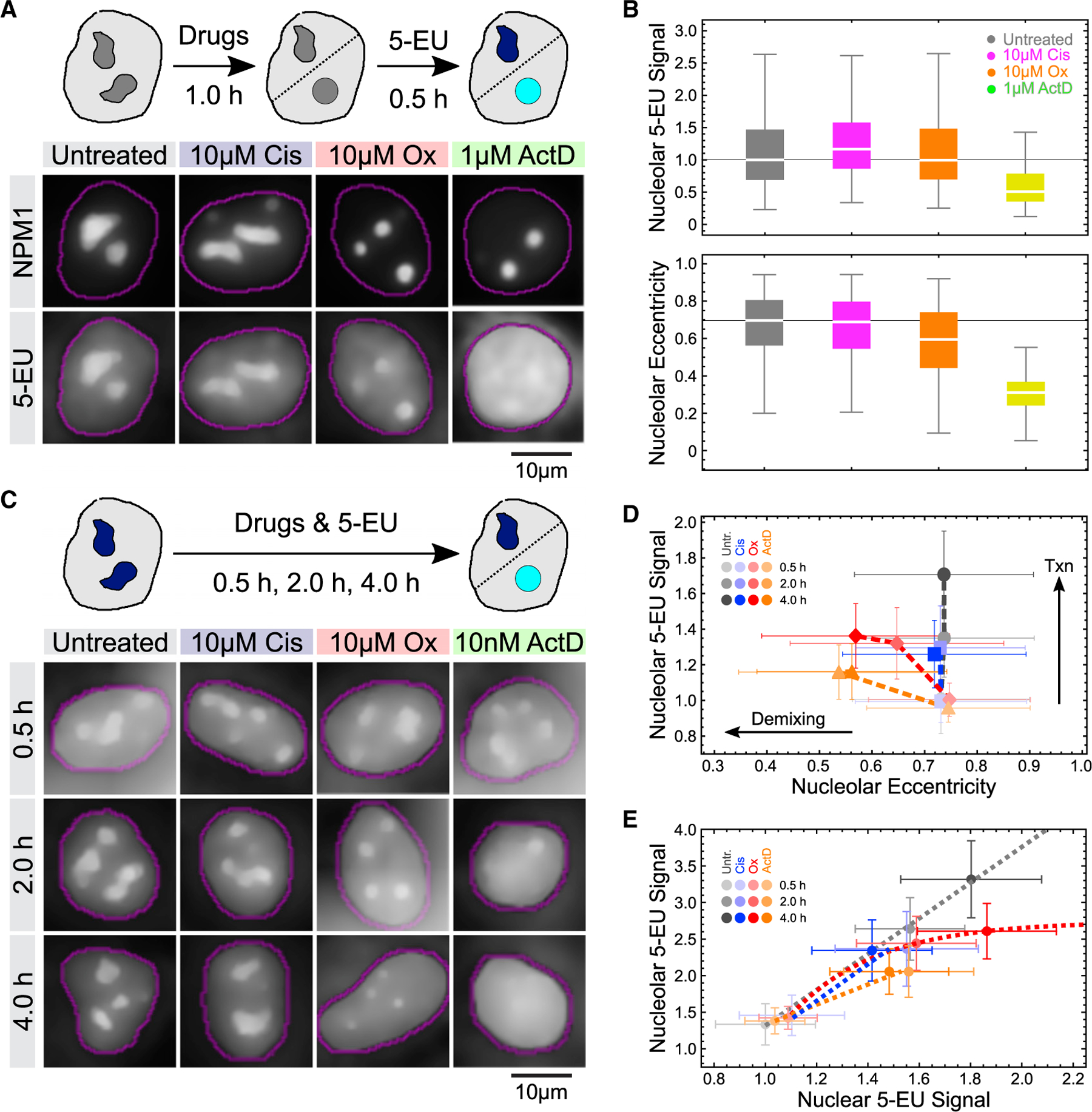
Oxaliplatin triggers a cycle of nucleolar demixing and transcriptional arrest (A) Scheme and representative epifluorescence images showing 5-EU incorporation into newly synthesized RNAs in U2OS cells after treatment with the indicated drugs. (B) Quantification of mean nucleolar 5-EU signals and nucleolar eccentricity in U2OS cells treated as in (A). Horizontal lines depict median values, boxes the 25th to 75th percentiles, and error bars the 5th to 95th percentiles. At least 713 nucleoli per condition were quantified. See Data S1 for statistics. (C) Scheme and representative epifluorescence images showing 5-EU incorporation in U2OS cells during drug treatments for the indicated times. (D) Quantification of changes in nucleolar eccentricity (x axis) and mean nucleolar 5-EU signals (y axis) over time in U2OS cells treated with the indicated drugs. 5-EU signals are normalized to the earliest nucleolar 5-EU measurement (30 min) in untreated cells. Color shades represent different time points. Dashed lines join data points to help visualization. Plot markers represent median values and error bars median deviation. At least 771 nucleoli per treatment and time point were quantified. See Data S1 for statistics. (E) Quantification of changes in nucleoplasmic (x axis) and mean nucleolar 5-EU signals (y axis) over time. Dashed lines indicated one-phase association fits for oxaliplatin and linear fits for untreated, cisplatin- and actinomycinD-treated U2OS cells. 5-EU signals are normalized to the earliest cytoplasmic 5-EU measurement (30 min) in untreated cells. Plot markers represent median values and error bars median deviation. At least 771 nucleoli per treatment and time point were quantified. See Data S1 for statistics.

If nucleolar demixing and shutdown of rRNA synthesis were sequential steps of oxaliplatin action, we predicted that there is a window where oxaliplatin alters nucleolar biophysics, but rRNA transcription is still ongoing in demixed nucleoli. Supporting this, cells treated with 10 μM oxaliplatin showed lower though still significant amounts of pre-rRNA (Figure 4E), suggesting active transcription. To further test this, we treated U2OS cells with oxaliplatin, cisplatin, and actinomycin D for an hour and monitored RNA transcription by pulse-labeling with the nucleotide analog 5-ethynyl uridine (5-EU), which can be covalently coupled to fluorophores after incorporation.^39^ As expected given the high nucleolar transcription rate, we observed a strong nucleolar 5-EU signal in untreated cells (Figures 5A and 5B). Strikingly, we detected a comparable nucleolar 5-EU signal (normalized to nucleolar area) in oxaliplatin-treated cells despite a decrease in nucleolar eccentricity, a quantitative yet facile readout for biophysical perturbation and demixing (Figure 5B). Note that because demixed nucleoli are smaller, total transcription levels are reduced in oxaliplatin-treated cells (Figure S5), a result consistent with previous findings.^9^ In contrast, actinomycin D both completely inhibited transcription and demixed nucleoli. Cisplatin slightly increased nucleolar rRNA levels, but did not affect nucleolar organization (Figures 5A and 5B). These data suggest that oxaliplatin-demixed nucleoli initially remain transcriptionally active.

To gain deeper insights into how oxaliplatin interferes with nucleolar form and function, we integrated transcriptional output over time (Figure 5C). In untreated cells, we detected a steady increase of nucleolar 5-EU signal over a 4 h period, while nucleolar morphology remained unchanged (Figures 5C and 5D). Oxaliplatin caused changes in nucleolar form but initially did not significantly affect the mean transcription rate per nucleolar area within the first 2 h. However, we observed a loss of transcriptional activity between 2 and 4 h, as well as further demixing (Figures 5C and 5D). In comparison, actinomycin D caused concurrent transcriptional shutdown and demixing. Even at a low dose (10 nM), at which actinomycin D selectively inhibits nucleolar Pol I (as evidenced by the 2 h time point), nuclear Pol II transcription was imparied after 4 h (Figure 5E), likely through indirect cellular toxicity. In contrast, cells treated with 10 μM oxaliplatin demonstrated robust inhibition of Pol I without affecting Pol II activity, even after 4 h (Figure 5E). Thus, oxaliplatin demonstrated more selectivity for Pol I than actinomycin D at later time points. Cisplatin affected neither nucleolar form nor function the first two hours, although we observed no further increase in 5-EU signal between 2 and 4 h (Figures 5C and 5D). This likely is a side effect of nucleolar-independent cisplatin toxicity, as nuclear RNA transcription is also affected (Figure 5E).

Together, our temporally resolved data demonstrate that the phenotype of oxaliplatin is distinct from a direct Pol I inhibitor, which first causes loss of nucleolar transcription that then leads to morphological defects in nucleoli. Instead, oxaliplatin causes defects in nucleolar morphology that either precede or happen concurrently with inhibition of rRNA synthesis. These observa suggest that oxaliplatin inhibits rRNA transcription indirectly.

### Oxaliplatin modifies nucleolar proteins *in vitro*

**Figure 6.**
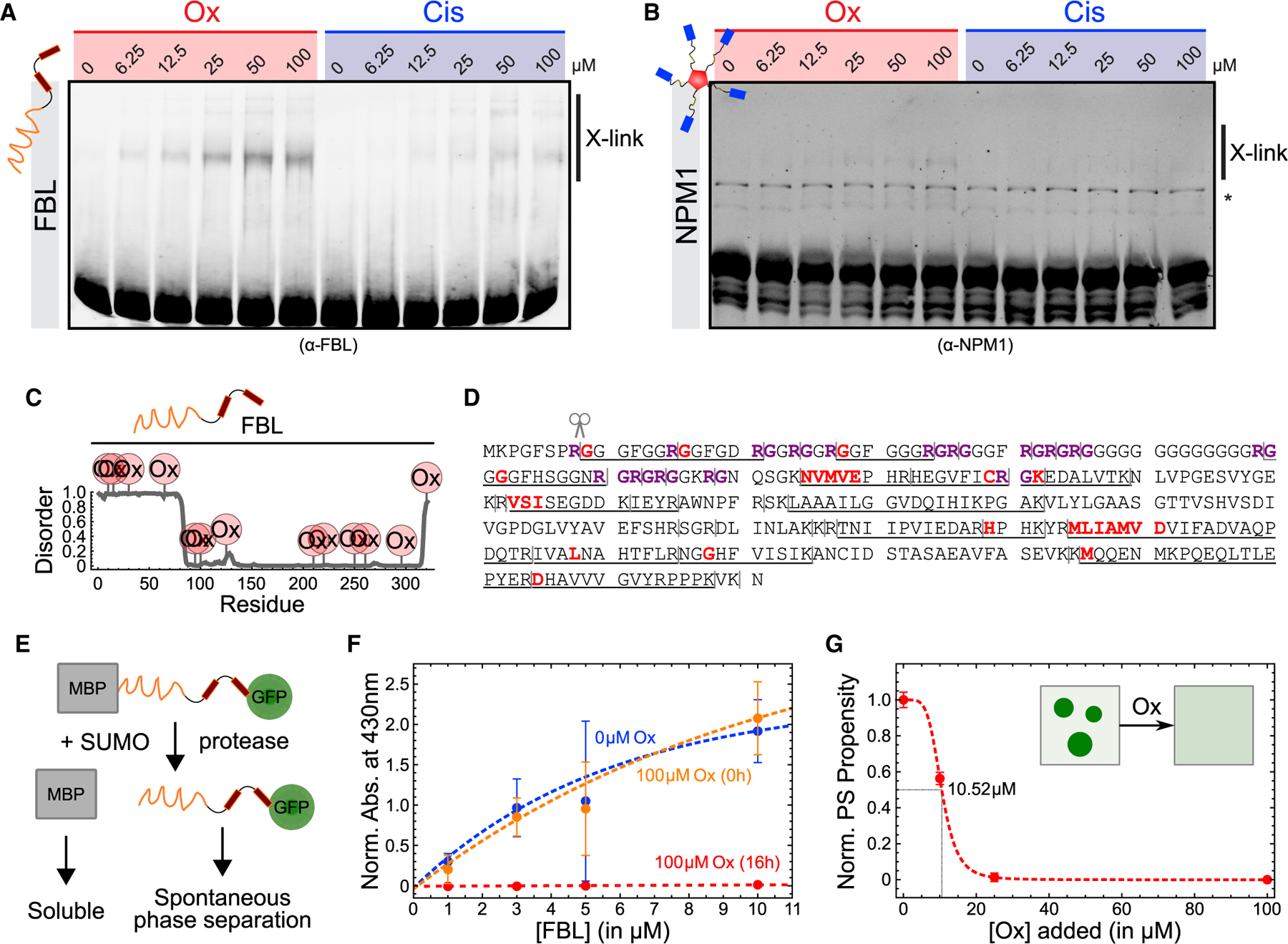
Oxaliplatin modifies FBL and alters its biophysical properties in vitro (A and B) Gel shift assays to detect the dose-dependent cross-linking of recombinant FBL (A) and NPM1 (B) by oxaliplatin (Ox) and cisplatin (Cis) visualized by immunoblotting. Labels mark platinated (+Pt) FBL and NPM1. Asterisks denote unspecific bands. Reactions were incubated for 20 h at room temperature. (C and D) Identification of platination sites on recombinant FBL by mass spectrometry after incubation with 100 μM oxaliplatin for 16 h. Identified sites were mapped onto disorder prediction of FBL (C) and primary amino acid sequence (D). Gray bars indicate predicted trypsin cleavage sites, black underscores identified peptides, bold red letters modified amino acids or motifs, and bold purple letters RG motifs. (E) Schematic of FBL *in vitro* phase separation assay. (F) Phase separation of unmodified (0 μM Ox) and oxaliplatin-modified (100 μM Ox, 16 h) FBL-GFP at various concentrations was monitored by measuring turbidity at 430 nm. As a control, 100 μM oxaliplatin was added only immediately before triggering phase separation (100 μM Ox, 0h). Plot markers represent mean values and error bars SD of triplicate measurements. (G) Phase separation propensity of FBL-GFP (at 10 μM) after modification with the indicated amounts of oxaliplatin for 16 h. Phase separation was monitored as in (H). Plot markers represent mean values and error bars SD of triplicate measurements.

Recent work showed that the formation and properties of multi-phase protein-RNA condensates such as nucleoli and stress granules are determined by the valency and stoichiometry of key nodes in the condensate interaction networks.^16,40–42^ Thus, such networks can be disrupted by removing central nodes or changing their valency. Pt compounds are known to modify and crosslink nucleic acids and proteins,^43^ suggesting that the chemical modification of nucleolar components with platinum may directly interfere with their valency. To test this model, we purified recombinant FBL and NPM1 from bacteria and incubated the proteins with varying concentrations of oxaliplatin and cisplatin. We found that oxaliplatin cross-linked FBL and, to a lesser extent, NPM1 in a dose-dependent manner, whereas cisplatin had little effect (Figures 6A and 6B). To assay the effect of oxaliplatin on nucleic acids, we mixed FBL and NPM1 with single-stranded RNA (ssRNA), single-stranded DNA (ssDNA), and double-stranded DNA (dsDNA) to form condensates that mimic the DFC and GC, respectively. We observed that oxaliplatin was less efficient at nucleic acid cross-linking, as evidenced by an immobilized nucleic acid population that could not enter the gel (Figures S6A and S6B). These findings suggest that compared with cisplatin, oxaliplatin preferentially targets and modifies FBL versus nucleic acids.

The striking morphological changes in nucleolar substructure *in vivo* and small extent of cross-linking *in vitro* suggest that oxaliplatin may introduce post-translational modifications (PTMs) that maintain (but alter) the overall liquid nature of nucleoli rather than stable intermolecular bonds that abolish nucleolar dynamics. To test this, we analyzed FBL treated with 100 μM oxaliplatin or cisplatin *in vitro* by liquid chromatography-mass spectrometry (LC-MS). We found that oxaliplatin modified FBL in the N-terminal intrinsically disordered region (IDR) and the surface-exposed loops of the folded domain (Figures 6C and S6C). Oxaliplatin reacted with different types of amino acids depending on the domain: in the IDR, it exclusively formed adducts with glycine residues, whereas it preferentially modified lysine and methionine residues in the loops (Figure 6D). We did not observe any FBL peptides with cisplatin modifications in our LC-MS analysis.

To test how oxaliplatination affects FBL phase separation under physiological conditions, we designed an *in vitro* phase separation assay that relies on the rapid removal of a solubilizing tag by protease cleavage to trigger spontaneous condensation, which then can be easily detected by measuring turbidity (Figure 6E). Unmodified FBL phase separated at concentrations as low as 1 μM, while FBL pre-treated with oxaliplatin remained soluble at concentrations up to 10 times higher (Figure 6F), with a dose-dependent effect (Figures 6G, S5C, and S5D). This was due to chemical modification rather than FBL solubilization or protease inhibition, as oxaliplatin had no effect on FBL phase separation when added concurrently with the protease (Figure 6E). Notably, the effect of oxaliplatin on FBL phase separation *in vitro* was consistent with its effect *in vivo*, where it decreased the driving force of FBL into nucleoli in two different cell types (unique from cisplatin and actinomycin D) (Figure 1H).

These results demonstrate that oxaliplatin forms adducts with side chains across all domains of FBL that modify its biophysical properties. We propose that this direct chemical modification can tune the valence of nucleolar proteins and thereby interfere with the nucleolar interaction network, similar as reported for phosphorylation, methylation, and other common PTMs.^44^

### Oxaliplatin toxicity can be alleviated by FBL overexpression

**Figure 7.**
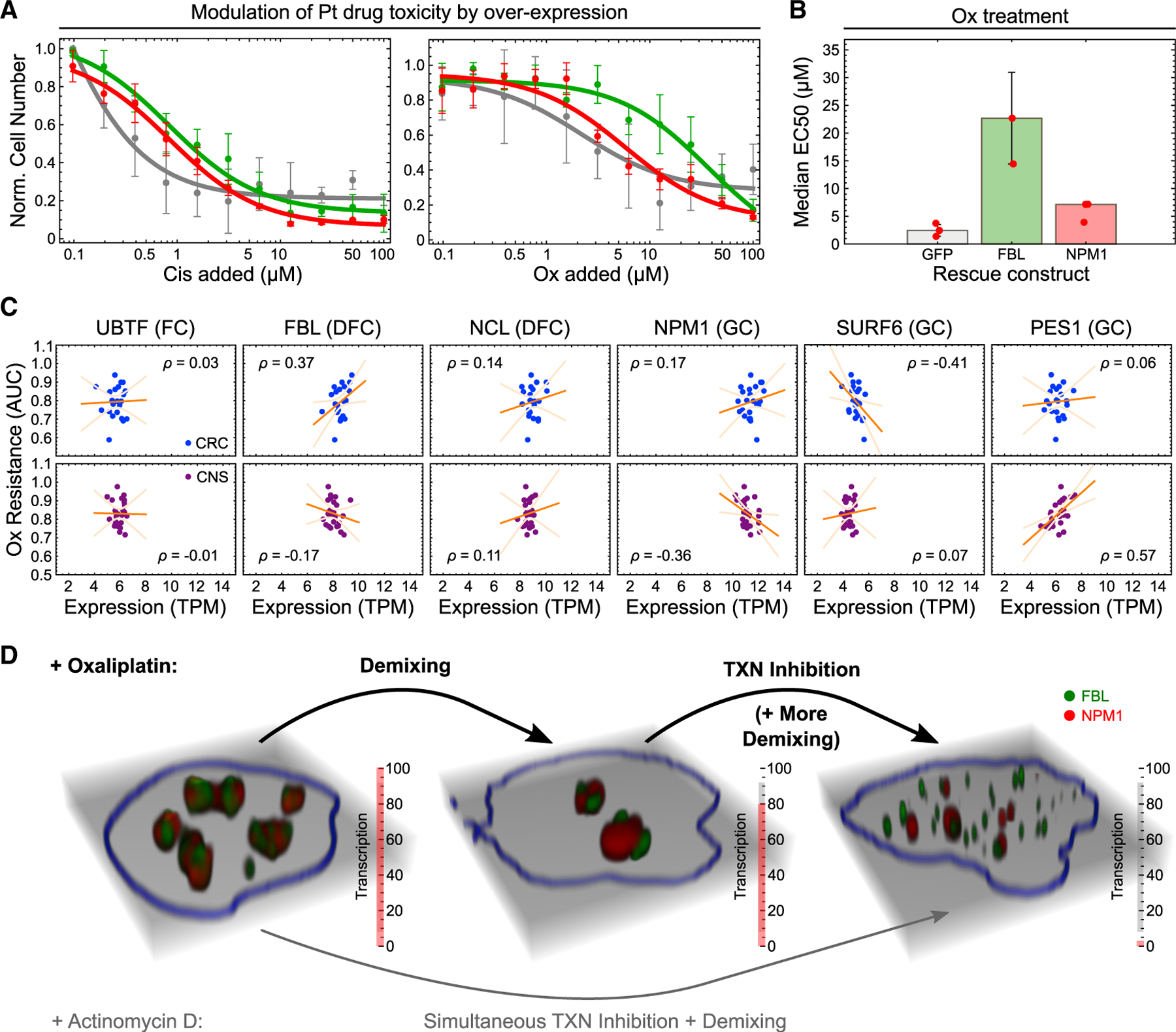
Oxaliplatin resistance correlates with FBL expression levels in colorectal cancer cells (A) Cell number versus drug concentration of HT-1080 cells transfected with either GFP, GFP-FBL, or GFP-NPM1, measured 48 h after treatment with the indicated amounts of Pt compounds. Data points represent the mean values from three replicates, error bars the SD, and curves a nonlinear dose-response fit to the data (see STAR Methods). (B) Comparison of EC_50_ values derived from the fits in (B) for oxaliplatin-treated HT-1080 cells rescued with the indicated constructs. Bar heights indicate median values, error bars median deviation and plot markers individual data points. See Data S1 for statistics. (C) Correlation between expression of the indicated nucleolar protein-coding transcripts (in TPM) and oxaliplatin resistance (quantified by the area under the dose-viability curve, AUC; see STAR Methods) in colorectal cancers (CRCs; blue) and central nervous system cancers (CNS; purple). Data points denote individual cancer cell lines, orange lines a linear fit to the data, light orange lines the 90% confidence interval of the fit, and ⍴ the Pearson correlation coefficient. See Data S1 for statistics. (D) Model summarizing the different mechanisms by which oxaliplatin and actinomycin D target nucleolar form and function.

Given our observation that FBL is modified by oxaliplatin in a dose-dependent manner, we sought to test how FBL overexpression affects cell growth in oxaliplatin-treated cells. To this end, we measured the toxicity of oxaliplatin and cisplatin in HT-1080 cells transfected with GFP-FBL, GFP-NPM1, or GFP alone. FBL overexpression conferred resistance to oxaliplatin, resulting in a >15-fold increase in half maximal effective concentration (EC_50_) (Figures 7A and 7B). This effect was specific to oxaliplatin and FBL, as it was not recapitulated with cisplatin or overexpression of NPM1 (Figure 7A).

To corroborate the relationship between FBL and oxaliplatin sensitivity, we analyzed the correlation between oxaliplatin resistance and transcript levels across 371 human cancer cell lines in the DepMap repository. Notably, FBL transcript levels correlated with oxaliplatin resistance in CRCs (Figure 7C). We neither observed this for other key nucleolar protein-coding transcripts, nor in other cancer types (Figures 7C and S7). Our findings show that FBL overexpression can alleviate oxaliplatin toxicity in cell culture models and suggest that this may be a strategy used by CRCs to increase oxaliplatin resistance.

## DISCUSSION

Our study suggests a plausible MOA explaining how oxaliplatin acts as a transcription and translation inhibitor: by chemically modifying key nucleolar scaffold components such as FBL, oxaliplatin causes the liquid-liquid demixing of nucleoli and triggers cell-cycle arrest. This biophysical defect then blocks transcription and impairs the function of nucleoli to supply cells with rRNA, which further demixes nucleoli, triggering a positive feedback loop that ultimately leads to cell death.

Our conclusions are based in part on phenotypic comparisons between oxaliplatin and the Pol I inhibitor actinomycin D that show distinct behaviors of oxaliplatin. In particular, we observe that oxaliplatin has a delayed effect on Pol I activity compared with its more immediate effect on morphology that is not observed with actinomycin D. This is consistent with oxaliplatin’s indirectly inhibiting Pol I via biophysical disintegration of nucleolar organization. It is also possible that the differences between oxaliplatin and actinomycin D may instead result because rRNA synthesis is inhibited more rapidly and effectively by actinomycin D than oxaliplatin. However, we observed delayed inhibition of rRNA synthesis even when oxaliplatin was administered at a 10 times greater dose than clinically relevant concentrations, making it unlikely that oxaliplatin directly inhibits Pol I.

Although this study does not identify direct molecular targets of oxaliplatin *in vivo*, four observations suggest that FBL may be a direct target. First, oxaliplatin has been shown to localize to nucleoli using nanometer-scale secondary ion mass spectrometry (nanoSIMS)-based imaging,^10^ suggesting that oxaliplatin co-localizes with FBL. Second, our *in vitro* data show that oxaliplatin can directly modify FBL and alter its phase separation (Figure 6). Third, oxaliplatin decreased the thermodynamic driving force for FBL phase separation in a dose-dependent manner across two different cell lines (Figure 1). Finally, overexpression of FBL alleviates oxaliplatin toxicity in both model cell lines and CRC cells (Figure 7). Given that oxaliplatin modifies FBL mostly at solvent-exposed residues in its IDR or loops (Figure S6), we consider it unlikely that there is a specific molecular recognition interaction between the two molecules. Rather, we suggest that oxaliplatin stochastically modifies nucleolar components with compatible reactive groups in its vicinity. This may include modulating additional pathways that affect nucleolar form and function in various stages of the cell cycle, including mTOR signaling pathway^18,45^ or the KI67-mediated anchoring of nucleoli in heterochromatin.^46^ As discussed below, the unbiased identification of oxaliplatin-modified nucleolar components *in vivo* remains a technical challenge that is beyond the scope of this work.

Our work demonstrates that oxaliplatin causes nucleolar compartments to demix yet maintain at least some of their scaffold components, allowing demixed nucleoli to remain transcriptionally active for some time and leaving Pol II activity unaffected. This may explain why oxaliplatin is able to effectively target ribosome biogenesis in the clinic, whereas the potent transcription inhibitor actinomycin D is too toxic for widespread use.^47^ Thus, targeting the biophysical properties of membrane-less organelles such as nucleoli may be a promising chemotherapeutic strategy, especially for translation-addicted cancers such as CRCs.^8,18^

The efficacy of oxaliplatin against gastrointestinal malignancies such as CRCs^48^ has been attributed to the unique translation addiction of colon cancer cells, which in turn makes them vulnerable to inhibition of ribosomal biogenesis by oxaliplatin.^8^ Our work suggests another, non-mutually exclusive model in which the biophysical properties of nucleoli in certain cancer cells may make them uniquely sensitive to oxaliplatin. Consistent with this model, abnormal nucleoli are a hallmark of cancer^49^ and recent work linked the chemical microenvironment of biomolecular condensates to the selective enrichment of small-molecule drugs with compatible physicochemical properties.^12,50^

We propose that the bulkiness and hydrophobicity of oxaliplatin allow it to preferentially enrich in nucleoli, where it then modifies proteins and potentially nucleolar RNAs. Given that nucleoli are organized in sublayers with different phase behavior,^25^ we consider it likely that oxaliplatin may cripple specific nucleolar compartments. Indeed, our observations that (1) oxaliplatin toxicity is rescued by FBL (DFC) but not NPM1 (GC) overexpression and that (2) rRNA transcription (FC) initially proceeds after treatment points toward the DFC as a preferential site for oxaliplatin action. Further deepening our understanding of how oxaliplatin modifications are able to rearrange nucleoli at the molecular and systems levels will help both unravel how oxaliplatin is specific to cancer cells and inform therapeutic strategies for targeting specific nucleolar regions and functions. Our findings demonstrate that expanding the arsenal of small molecules targeting biomolecular condensates is a promising strategy for developing the next generation of antineoplastic drugs.^51^

### Limitations of the study

A major barrier to uncovering the MOA of orphan drugs such as oxaliplatin is the lack of imaging modalities to accurately and quantitatively determine their subcellular localization. Although nanoSIMS imaging shows an enrichment of oxaliplatin in nucleoli,^10^ fluorescence-based methods would increase spatial and temporal resolution of drug localization. Fluorescent versions of oxaliplatin have been generated, for example by tethering it to lipophilic fluorescent dyes.^52^ However, these tools significantly change the physicochemical properties of oxaliplatin. Although the latter often is acceptable for modified drugs that retain high binding affinity for their specific targets, it is problematic for drugs that selectively partition into condensates and membrane-less organelles on the basis of their physicochemical properties.^50,51^ Indeed, uncovering the chemical code that specifies how small-molecule drugs target condensates is an emerging frontier at the intersection of chemical biology and cellular biophysics that has the potential to inform novel avenues to drug discovery.

A second technical challenge is the unbiased identification of nucleolar proteins that are modified by oxaliplatin, for three reasons that greatly complicate mass spectrometry work flows. First, many key nucleolar proteins contain extensive IDRs that typically lack trypsin cleavage sites, which either results in reduced protein coverage or requires the use of bespoke protease cocktails. Second, our work suggests that oxaliplatin can form adducts with a variety of amino acid side chains, further increasing the complexity of possible peptide species. Third, sampling depth and detection sensitivity can be limiting, especially when analyzing complex samples such as nucleolar or nuclear extracts. An ideal solution for these problems would be the development of an affinity matrix that enriches oxaliplatin-modified peptides, similarly as in phosphoproteomics.

## STAR★METHODS

### RESOURCE AVAILABILITY

#### Lead contact

Further information and requests for resources and reagents should be directed to and will be fulfilled by the lead contact Onn Brandman (onn@stanford.edu).

#### Materials availability

All unique/stable reagents generated in this study are available from the lead contact without restriction.

#### Data and code availability

Imaging raw data are publicly available on Zenodo (see key resources table for DOIs).

Custom scripts for data processing and analysis are publicly available on Github (https://github.com/RohatgiLab/2022_Schmidt_Oxaliplatin).

Any additional information required to reanalyze the data reported in this paper is available from the lead contact upon request.

### EXPERIMENTAL MODEL AND SUBJECT DETAILS

#### Cell culture

HCT116 (ATCC CCL-247) and U2OS (ATCC HTB-96) cell lines were maintained at 37°C and 5% CO_2_ in DMEM high glucose (GE Healthcare) supplemented with 10% FBS (Atlanta Biologicals), 2mM L-glutamine (Gemini Biosciences), 1mM sodium pyruvate (Gibco), 1x MEM non-essential amino acids solution (Gibco), 40 U/mL penicillin and 40 μg/mL streptomycin (Gemini Biosciences).

HT-1080 (ATCC CCL-121) cells were grown in DMEM high glucose medium (Corning Life Science) supplemented with 1% non-essential amino acids (Life Technologies). HT-1080 cells used in this study were stably infected with Nuc:mKate2, a red fluorescent protein targeted to the nucleus.^56^

### METHOD DETAILS

#### Generation of knock-in cell lines

Genome engineering of HCT116 and U2OS cells was performed either by (a) co-transfection of plasmids encoding for Cas9, sgRNAs and homology donors or (b) co-electroporation of preformed Cas9/sgRNA ribonucleo-protein (RNP) complexes and double-stranded homology template donors. Plasmid-based homology donors contained unique, synthetic sgRNA cutting sites 5′ and −3′ of the donor construct for linearization in cells (see key resources table). Cas9/sgRNA RNPs were assembled *in vitro* as described.^57^ Homology donor sequences (see key resources table) were cloned from genomic DNA or obtained by PCR amplification as in^58^ for the plasmid- and RNP-based methods, respectively. To enhance homologous recombination during RNP-based editing, U2OS cells were first synchronized by treatment with 200 ng/mL nocodazole for 20 h.^55^ Synchronized cells were harvested from the culture supernatant and electroporated in 96-well format using an Amaxa Shuttle nucleofection device (CM-104 program, Lonza). For each individual electroporation, 200,000 cells resuspended in SE solution were mixed with 10 pmole Cas9/sgRNA RNP and 5 pmole homology template. Successfully edited cells by either method were selected by fluorescence-activated cell sorting (FACS) on a SONY SH800 instrument.

#### Fluorescence imaging of nucleolar organization

Unless otherwise specified, HCT116 and U2OS knock-in cells were seeded at 15,000 cells/well in 18-well ibidi glass-bottom μ-slides, allowed to adhere o/n, treated as indicated and imaged with an Olympus IX83 epifluorescence microscope equipped with an Orca Fusion scMOS camera and a 100× oil objective (NA 1.45). At least 15 z planes in 0.26 μm steps were taken per position and channel. Images were deconvoluted with the Olympus cellSense software using the proprietary constrained iterative deblurring algorithm.

#### Estimation of NPM1 surface tension

U2OS knock-in cells were seeded at 15,000 cells/well in an 18-well ibidi glass-bottom μ-slide and allowed to adhere o/n. After treatment with the indicated amounts of drugs for 4 h, the cells were imaged with an Olympus IX83 epifluorescence microscope equipped with an Orca Fusion scMOS camera, a 100× oil objective (NA 1.45), an X3-ZDC2 TruFocus drift compensator and an Okolab IX3-SVR stage top incubator set to 37°C and 5% CO2. 100 consecutive frames in the FITC channel were taken per field of view at 250 ms intervals and analyzed as previously described^26^ using custom Mathematica scripts. In short, the surface fluctuation u of individual nucleoli was quantified by averaging the changes in nucleolar contour over both time and polar angle using the Interpolation and Fourier transformation functions of Mathematica. The average surface fluctuation $\langle u\rangle$ was then used to estimate nucleolar surface tension γ based on the relation $\gamma ={\text{k}}_{\text{B}}\text{T}/\langle u^{2}\rangle$.

#### Estimation of ∆G^tr^ and ∆∆G^tr^ for NPM1 and SURF6

15,000 HCT116 or U2OS knock-in cells were seeded per well of a 18-well ibidi glass-bottom μ-slide and allowed to adhere o/n. Cells were then treated with the indicated amounts of drugs for 4 h, fixed with 4% PFA for 15 min at room temperature, and either immunostained for SURF6 (as described below) or imaged directly. Following washing, cells were kept in PBS and imaged using an Olympus IX83 epifluorescence microscope equipped with an Orca Fusion scMOS camera, a 40× oil objective (NA 1.4) and an X3-ZDC2 TruFocus drift compensator. For every well, a 9 × 9 grid was automatically acquired in the FITC and TRITC channels.

The free energy for the transfer of NPM1 and SURF6 from the dilute phase (nucleoplasm) to the dense phase (nucleoli) was estimated as previously described.^16^ In short, the image segmentation and quantification functions of Mathematica were used to quantify the nucleoplasmic and nucleolar signals, I^dilute^ and I^dense^, for both NPM1 and SURF6 (Fig. S1C). These values were then used to determine the partitioning coefficient *K* = I^dense^/I^dilute^ and the free energy of transfer ∆G^tr^ = − *RT* ln*K*.

#### Immunofluorescence stainings and imaging

HCT116 or U2OS knock-in cells were seeded onto acid-washed #1.5 glass coverslips (at a density of 50,000 cells/coverslip). Following treatment with the indicated amounts of drugs, cells were fixed with 4% PFA for 15 min at room temperature and permeabilized in blocking buffer (PBS supplemented with 0.5% Triton X-100, 1% donkey serum and 10 mg/mL BSA) for 30 min at room temperature. Cells were then incubated with primary antibodies (key resources table; diluted 1:500 in blocking buffer) for 16 h at 4°C. After washing three-times with PBS containing 0.2% Triton X-100, cells were further incubated with Alexa 647-labeled secondary antibodies (key resources table; diluted 1:500 in blocking buffer) for 1 h at room temperature. Following three washes with PBS containing 0.2% Triton X-100, cells were mounted onto microscopy slides with ProLong Diamond mounting medium containing DAPI (Molecular Probes).

Unless otherwise specified, cells were imaged using a Leica SP8 laser-scanning confocal microscope equipped with a 63x (1.4 NA) oil immersion objective. Images were analyzed and quantified using custom Mathematica scripts.

#### Cell synchronization and live cell imaging

1 × 10^6^ U2OS knock-in cells were seeded in a 10cm culture dish and incubated for 24 h. The culture medium was then replaced with fresh DMEM containing 2mM thymidine. After 18 h, cells were washed twice with PBS, allowed to recover for 6 h in fresh DMEM, and subjected to a second block with 2mM thymidine for 18 h.

Synchronized cells were then trypsinized, seeded into 8-well glass-bottom μ-slides (ibidi) at a density of 30,000 cells/well, and allowed to adhere for three hours. Culture medium was then exchanged with L-15 medium (Gibco) supplemented with 10% FBS and the indicated amounts of drugs. Cells were imaged every 15 min for a total of 24 h in a 3 × 3 grid per well using an Olympus IX83 epifluorescence microscope equipped with an Orca Fusion scMOS camera, a 403 air objective (NA 0.95), an X3-ZDC2 TruFocus drift compensator and an Okolab IX3-SVR stage top incubator set to 37°C. Images were quantified using custom Mathematica scripts.

To determine cell cycle stage, samples were taken immediately after synchronization and at the time point of drug addition, fixed and permeabilized with ice-cold 70% ethanol at 4°C overnight, stained with 1 μg/mL propidium iodine in PBS containing 0.1% Triton X-100 and 10 μg/mL RNase, and analyzed using a Sony SH800 flow cytometer.

#### Quantification of rRNA levels by RT-PCR

U2OS knock-in cells were treated with the indicated amounts of cisplatin, oxaliplatin and actinomycin D for 4 h. Total RNA was extracted using the Trizol reagent (Ambion) according to the manufacturer’s instructions and transcribed into cDNA using the iScript kit (Bio-Rad). To measure rRNA levels, the Power SYBR Green kit (Applied Biosystems) was used for quantitative RT-PCR analysis with primers against the 5′ ETS of the 45S pre-rRNA, the 18S and 28S rRNAs, and GAPDH (see key resources table for sequences). The delta-delta-Ct method was used for normalization (relative to GAPDH levels) and fold-change calculation (relative the untreated condition).

#### RNA-FISH staining and imaging

U2OS knock-in cells were seeded onto acid-washed #1.5 glass coverslips (at a density of 50,000 cells/coverslip). After 24 h, the cells were treated for 0, 0.5, 1, 2 and 4 h with the indicated amounts of drugs in DMEM medium. After fixation with 4% PFA for 15 min at room temperature, cells were washed with PBS and incubated in 70% ethanol for 16–24 h at 4°C. Cells were then washed with 2x SSC buffer (Ambion) containing 10% formamide (Ambion) and stained with 125 nM dye-labeled FISH probes (Stellaris; see key resources table for sequences) in 2x SSC buffer (Ambion) containing 10% formamide (Ambion) and 10% (w/v) dextran sulfate (Sigma) for 16 h at 37°C in a humidity chamber. After two washes with 2x SSC buffer (Ambion) containing 10% formamide (Ambion) for 30 min at 37°C each, cells were mounted onto microscopy slides with ProLong Diamond mounting medium containing DAPI (Molecular Probes). All solutions for RNA-FISH were prepared with nuclease-free water (Ambion).

Stained cells were imaged using a Leica SP8 laser-scanning confocal microscope equipped with a 63x (1.4 NA) oil immersion objective and quantified using custom Mathematica scripts. For 3D reconstructions, z-stacks (5.53 μm total in 0.221 μm steps) of selected cells were taken on an Olympus IX83 epifluorescence microscope equipped with an Orca Fusion scMOS camera and a 100× oil objective (NA 1.45). Stacks were then deconvoluted as described above and processed using custom Mathematica scripts.

#### 5-Ethynyl uridine (5-EU) incorporation and click labeling

To label nascent RNA, 1 mM 5-EU (Thermo Fisher) was added to U2OS knock-in cells grown on #1.5 glass coverslips, either following or during drug treatments as indicated. In case 5-EU incorporation occurred after drug treatments, cells were incubated for 30 min at 37°C and 5% CO_2_. Cells were then fixed with 1% PFA in PBS for 20 min at room temperature, washed three-times with PBS +0.05% Tween 20, and permeabilized with PBS +0.5% Triton X-100. Following blocking with PBS +10% fetal bovine serum (Atlanta Biologicals), incorporated 5-EU was labeled with Alexa Fluor 647 azide (Invitrogen) via copper-catalyzed click chemistry. To this end, click reaction cocktail was prepared by adding 5 μM dye, 0.5 mg/mL copper sulfate and 20 mg/mL freshly resuspended sodium ascorbate to PBS, and added to cells for 30 min at room temperature in the dark. Finally, the coverslips with the cells were washed three-times with PBS +0.05% Tween 20 and mounted onto glass slides with ProLong Glass NucBlue (Invitrogen).

#### Recombinant protein purification

Recombinant FBL and NPM1 were expressed and purified essentially as described previously.^25^ Both FBL and NPM1 were cloned and expressed using the pET system in E. coli BL21(DE3) Rosetta. Transformed cells were grown in LB at 37°C to an OD600 of 0.6 before inducing with 0.5 mM IPTG. Expression continued for up to 4 h before cells were pelleted and frozen before protein purification.

FBL-expressing cell paste was resuspended in buffer containing 20 mM Tris-HCl, pH 7.5, 500 mM NaCl, 10 mM imidazole, 14 mM β-mercaptoethanol, 10% (vol/vol) glycerol and a protease inhibitor mixture (Roche Diagnostics). FBL was purified first over a Ni^2+^-NTA column and by subsequent purification over heparin resin and polished by size exclusion chromatography. FBL was stored at 10 mg/mL in 20 mM Tris, pH 7.4, 1 M NaCl, 1% (vol/vol) glycerol, and 2 mM DTT.

NPM1-expressing cell paste was resuspended and lysed by sonication in buffer containing 20 mM Tris, 300 mM NaCl, 10 mM β-mercaptoethanol, 20U/mL Benzonase (Millipore), and a protease inhibitor mixture (Roche Diagnostics). NPM1 was purified first over a Ni^2+^-NTA column subsequently by anion exchange and polished by size exclusion chromatography. NPM1 was stored at 5 mg/mL in 10 mM Tris, 0.15 M NaCl, 2 mM DTT, pH 7.5.

MBP-SUMO-FBL-GFP with a C-terminal, TEV-cleavable His16 tag was expressed in *E. coli* NEB Express cells overnight at room temperature following induction with 50 μM IPTG. Pelleted bacteria were resuspended in IMAC buffer (50 mM Tris pH8, 2 M NaCl, 4.4 mM MgCl_2_, and 1mM PMSF) and flash-frozen in liquid nitrogen. For lysis, bacteria were thawn, supplemented with 1 mM DTT and 25 mM imidazole, and sonicated on ice. Lysates were cleared by ultracentrifugation and bound to Ni^2+^-NTA agarose beads (Gold Bio). Buffer was exchanged on-column to LS buffer (44 mM Tris pH7.4, 290 mM NaCl, 4.4 mM MgCl_2_ and 1 mM DTT) prior to elution with LS buffer containing 400 mM imidazole. The His16 tag was then cleaved off with TEV protease overnight, the imidazole removed using a PD10 gel-filtration column (Cytiva) equilibrated with LS buffer containing 250 mM sucrose, and all uncleaved protein removed on a reverse Ni^2+^ affinity column. Purified protein was aliquoted, flash-frozen in liquid nitrogen and stored at −80°C until further use.

#### *In vitro* platination assays

Single-use aliquots of FBL were thawed on ice and diluted to 1 mg/mL in 20 mM Tris-HCl pH 7.4, and 100 mM NaCl in the presence or absence of 100 μg/mL nucleic acid and platinum compounds. Similarly, NPM1 platination assays were performed by diluting single-use aliquots of NPM1 to 0.5 mg/mL in 10 mM Tris pH 7.5, 0.15 M NaCl, and 2 mM DTT, as well as 100 μg/mL nucleic acids where indicated. Platination of nucleolar proteins and/or nucleic acids was carried out at 25°C for 16–24 h in the dark. Total RNA was prepared by phenol-chloroform extraction from HeLa cell pellets. rRNA was purchased from BioWorld. ssDNA source was 20/100 oligo length ladder (IDT), ssRNA source was RiboRuler Low Range RNA standard (Thermo Fisher) and dsDNA ladder was 1kb Plus DNA ladder (NEB).

Protein and nucleic acid platination was assessed by gel shift assays using SDS-PAGE (protein), standard AGE (dsDNA), urea-AGE (ssDNA) and HEPES/triethanolamine-formaldehyde AGE (ssRNA). Proteins were detected by immunoblotting using antibodies against FBL and NPM1 (see key resources table). Nucleic acids were stained with SYBR Green.

#### Mass spectrometry analysis of *In vitro* platinated nucleolar proteins

FBL and NPM1 were incubated with 100 μM cisplatin or oxaliplatin for 16 h at room temperature in the absence of nucleic acids, as described above. Samples were then mixed with trypsin, adjusted to ~0.02 mg/mL and incubated at 37°C for 4 h before immediate stage tipping and LC-MS/MS analysis. To preserve potential fragile modifications, samples were analyzed both with and without additional reduction and alkylation for 2 h at 37°C with 0.5 mM DTT and 1.5 mM iodoacetamide, respectively. LC was done with a C18 nanoLC column on an Eksigent Ekspert nanoLC 400 running a 30-min water-to-acetonitrile gradient with 0.1% formic acid. Positive nanoESI introduced the sample to a Thermo Orbitrap Elite that acquired MS1 and MS2 scans by orbitrap. As the oxaliplatin and cisplatin might have modified the samples in unexpected ways, in-house software determined the xCorr score of the match between each possible peptide and each MS2 with any one residue allowed a modification with a mass equal to the delta between the calculated mass of the unmodified peptide and the observed mass of the MS2 precursor peaks.

#### FBL phase separation assays

MBP-SUMO-FBL-GFP was adjusted to the indicated concentrations, diluted to 20 mM Tris pH7.4, 130 mM NaCl, 2 mM MgCl_2_ with ddH_2_O and treated with 0, 10, 25, 50 or 100 μM oxaliplatin for 16 h at room temperature in the dark. Spontaneous phase separation was triggered by addition of 50 nM SUMO protease and monitored (i) by measuring absorbance at 430 nm on a Synergy H1 plate reader (BioTek) and (ii) on a 10% denaturing polyacrylamide gel after spinning the samples at max. speed in an Eppendorf table-top microfuge for 30 min.

#### Drug toxicity rescue assays

To determine the impact of NPM1 or FBL overexpression on Cis and Ox-induced lethality, HT-1080 Nuc:mKate2 cells were transfected with constructs overexpressing either GFP-NPM1, GFP-FBL, or GFP alone. Briefly, 90,000 cells were seeded into a well of a 6-well plate. Once adherent, media was replaced with antibiotic free growth media for 20 min. Then, cells were incubated in a mixture of OptiMEM (ThermoFisher), Lipofectamine LTX (ThermoFisher), and recombinant DNA as per manufacturer protocol. 48 h after transfection, the cells were lifted and seeded into 384-well plates at a density of 1500 cells/well. The next day, the growth media was changed to fresh media containing cisplatin or oxaliplatin, dosed in a two-fold dilution for a ten-point dose-response curve where the highest dose was 100 μM.

Cell viability was then analyzed using the IncuCyte imaging platform (Sartorius), as previously described.^56^ Briefly, the assay measures live cells based on nuclear mKate signals. Images were captured in the red fluorescent mKate channel at 10× magnification every 4 h for a total of 48 h post drugging. To count cells, image segmentation was performed using MATLAB. Total cells were counted based on threshold for mKate2 levels and transfected cells were counted based on threshold for GFP levels. EC_50_ values were determined as for the MTT assay, except that the concentration-dependent cell number was fitted instead of the MTT intensity.

#### Cancer transcriptomics and drug resistance analysis

The DepMap portal (https://depmap.org) was used to obtain the PRISM high-throughput drug screen^59^ and CCLE expression^60^ datasets. Across the 371 cancer cell lines covered by both datasets, the Pearson correlation coefficient between oxaliplatin resistance (expressed as the area under the dose-viability-curve, AUC) and expression levels was calculated for 19,177 transcripts (in TPM) using custom Python scripts. The ShinyGO web server (https://bioinformatics.sdstate.edu/go/) was used for GO term analysis. To compare oxaliplatin-sensitive and -insensitive cancers, correlation analysis was performed separately for 23 colorectal cancer and 25 central nervous system cancer cell lines, respectively. For select nucleolar transcripts, oxaliplatin resistance was plotted against transcript levels and fitted with a simple linear regression model to visualize correlation trends.

#### MTT assay and dose-response determination

1 × 10^4^ synchronized cells were seeded per well of two 96-well plates and allowed to adhere for three hours. Cells were then treated with 0, 0.5, 1, 2.5, 5, 7.5, 10, 25, 50, 75, 100, and 200 μM oxaliplatin or cisplatin (four replicates per condition). After 24 and 48 h, 0.22 μg/mL MTT reagent (3-(4,5-dimethylthiazol-2-yl)-2,5-diphenyltetrazolium bromide) was added per well. After incubation at 37°C and 5% CO_2_ for three hours, the culture medium was removed and cells were solubilized in 50 μL DMSO per well for 5 min at 37°C and 5 min at room temperature. Absorbance (Abs) of the solubilized MTT dye was then quantified at 570 nm and 690 nm using a Biotek Synergy HT microplate reader. For analysis, the MTT intensity *I* at concentration *c* was defined as *I(c)* = *Abs*_*570nm*_*(c)* – *Abs*_*690nm*_*(c)*, and *I(c)* plotted against *c*. To determine EC_50_ values, the data were fitted to the equation:, where *I(c*_*0*_*)* is the maximal MTT intensity, *I(c*_*min*_*)* the minimal MTT intensity and *n* = 2.

### QUANTIFICATION AND STATISTICAL ANALYSIS

Mathematica (Wolfram Research) was used for statistical testing. Information on tests and statistics can be found in the figure legends and Data S1.

## Supplementary Material

MMC1

MMC2

## Figures and Tables

**Table:** KEY RESOURCES TABLE

Reagent and resource	Source	Identifier
Antibodies		
Rabbit ɑ-NPM1 polyclonal antibody	Santa Cruz	sc-5564
Rabbit ɑ-FBL monoclonal antibody	Cell Signaling	2639
Rabbit ɑ-SURF6 polyclonal antibody	Atlas/Sigma	HPA023608
Rabbit ɑ-MKI67 polyclonal antibody	Atlas/Sigma	HPA000451
ɑ-Rabbit-Alexa488 polyclonal antibody	Invitrogen	A-21206
ɑ-Rabbit-Alexa647 polyclonal antibody	Invitrogen	A-31573
ɑ-Rabbit-IR800 polyclonal antibody	LiCor	926-49020
Bacterial and virus strains		
*E. coli* TOP10	Invitrogen	C404010
*E. coli* NEB Express *I*^*q*^	New England Biolabs	C3037I
*E. coli* BL21(DE3) Rosetta	Novagen	70954
Chemicals, peptides, and recombinant proteins		
Oxaliplatin	Acros Organics	AC456131000
Cisplatin	Biotang	RYG01
Actinomycin-D	MP Biomedicals	02194525-CF
5-ethynyl uridine (5-EU)	Thermo Fisher	E10345
AlexaFluor 647 azide	Thermo Fisher	A10277
Recombinant FBL	Home-made	^25^
Recombinant NPM1	Home-made	^25^
Recombinant MBP-bdSUMO-FBL-GFP	Home-made	This study
Recombinant TEV protease	Home-made	^53^
Recombinant bdSENP1	Home-made	^54^
Recombinant Cas9	Home-made	^55^
Critical commercial assays		
iScript cDNA Synthesis Kit	Bio-Rad	1708891
Power SYBR Green PCR Master Mix	Applied Biosystems	4367659
Deposited data		
DS1_Morphometry_RawData	This study (Figure 1)	10.5281/zenodo.6643187
DS2_SurfaceTension_RawData	This study (Figure 2)	10.5281/zenodo.6643398
DS3a_Thermodynamics_HCT116_RawData	This study (Figures 1 and 2)	10.5281/zenodo.6643525
DS3b_Thermodynamics_U2OS_RawData	This study (Figures 1 and 2)	10.5281/zenodo.6643587
DS4_KI67_RawData	This study (Figure 3)	10.5281/zenodo.6643649
DS5_FISH_RawData	This study (Figure 4)	10.5281/zenodo.6643654
DS6a_5EU_Pulse_RawData	This study (Figure 5)	10.5281/zenodo.6643470
DS6b_5EU_Time Course_RawData	This study (Figure 5)	10.5281/zenodo.6643490
Experimental models: Cell lines		
Human HCT116 FBL-GFP NPM1-RFP knock-in cells	This study	N/A
Human U2OS FBL-GFP NPM1-RFP knock-in cells	This study	N/A
Human U2OS UBTF-GFP FBL-Halo NPM1-RFP knock-in cells	Leonetti Lab	czML0501
Human HT-1080 Nuc::mKate2	Dixon Lab	^56^
Oligonucleotides		
45S pre-rRNA_qPCR_fwd Gccgtgcctgaggtttct	ELIM Biopharm	N/A (custom)
45S pre-rRNA_qPCR_rev Accaacggacgtgaagcc	ELIM Biopharm	N/A (custom)
18S_qPCR_fwd Ctggataccgcagctaggaa	ELIM Biopharm	N/A (custom)
18S_qPCR_rev Gaatttcacctctagcggcg	ELIM Biopharm	N/A (custom)
28S_qPCR_fwd Cggcgggagtaactatgact	ELIM Biopharm	N/A (custom)
28S_qPCR_rev gctgtggtttcgctggatag	ELIM Biopharm	N/A (custom)
GAPDH_qPCR_fwd aaagggtcatcatctctg	ELIM Biopharm	N/A (custom)
GAPDH_qPCR_rev gctgttgtcatacttctc	ELIM Biopharm	N/A (custom)
45S pre-rRNA_Probe1-Quasar 670nm gacacgcacggcacggag	Stellaris	N/A (custom)
45S pre-rRNA_Probe2-Quasar 670nm ccgcggagacgagaacgc	Stellaris	N/A (custom)
45S pre-rRNA_Probe3-Quasar 670nm ggaaggggcggcggacaa	Stellaris	N/A (custom)
45S pre-rRNA_Probe4-Quasar 670nm cgggagagcacgacgtca	Stellaris	N/A (custom)
45S pre-rRNA_Probe5-Quasar 670nm gttcgccacgaacgtccg	Stellaris	N/A (custom)
45S pre-rRNA_Probe6-Quasar 670nm cggagcgagaaggacggt	Stellaris	N/A (custom)
45S pre-rRNA_Probe7-Quasar 670nm tctgccgcgtcagaggac	Stellaris	N/A (custom)
45S pre-rRNA_Probe8-Quasar 670nm cgcccgcaagtcgacaac	Stellaris	N/A (custom)
45S pre-rRNA_Probe9-Quasar 670nm cgagagggcagcacgacg	Stellaris	N/A (custom)
45S pre-rRNA_Probe10-Quasar 670nm agccgacgctcgcgcaaa	Stellaris	N/A (custom)
45S pre-rRNA_Probe11-Quasar 670nm ctccaggagcaccgcaag	Stellaris	N/A (custom)
45S pre-rRNA_Probe12-Quasar 670nm ctgagggacaacccggag	Stellaris	N/A (custom)
45S pre-rRNA_Probe13-Quasar 670nm gaacgacacaccaccgtt	Stellaris	N/A (custom)
45S pre-rRNA_Probe14-Quasar 670nm gacgagctccctcaggac	Stellaris	N/A (custom)
45S pre-rRNA_Probe15-Quasar 670nm tcaaaccgcctcgaaccc	Stellaris	N/A (custom)
45S pre-rRNA_Probe16-Quasar 670nm cagaggggagcacgggac	Stellaris	N/A (custom)
45S pre-rRNA_Probe17-Quasar 670nm caccgcgatcgctcacac	Stellaris	N/A (custom)
45S pre-rRNA_Probe18-Quasar 670nm tcggaggcagaacggcag	Stellaris	N/A (custom)
45S pre-rRNA_Probe19-Quasar 670nm tccgaagtcaacccacac	Stellaris	N/A (custom)
45S pre-rRNA_Probe20-Quasar 670nm tcgagcgttcgcgttcag	Stellaris	N/A (custom)
45S pre-rRNA_Probe21-Quasar 670nm cgaggaaacacctgcgcg	Stellaris	N/A (custom)
45S pre-rRNA_Probe22-Quasar 670nm cttttctcaccgagggtg	Stellaris	N/A (custom)
45S pre-rRNA_Probe23-Quasar 670nm cctctcagatcgctagag	Stellaris	N/A (custom)
45S pre-rRNA_Probe24-Quasar 670nm acggcagcgctaccataa	Stellaris	N/A (custom)
45S pre-rRNA_Probe25-Quasar 670nm cacagtaggcgacgagcc	Stellaris	N/A (custom)
FBL_HDR_sgRNA_fwd caccgAACTGAAGTTCAGCGCTGTC	ELIM Biopharm	N/A (custom)
FBL_HDR_sgRNA_rev aaacGACAGCGCTGAACTTCAGTTc	ELIM Biopharm	N/A (custom)
NPM1_HDR_sgRNA_fwd caccgGCCAGAGATCTTGAATAGCC	ELIM Biopharm	N/A (custom)
NPM1_HDR_sgRNA_rev aaacGGCTATTCAAGATCTCTGGCc	ELIM Biopharm	N/A (custom)
Recombinant DNA		
pHBS1707 CtaggerV6-FBL-sfGFP	This study	https://benchling.com/s/seq-f7NxUgzlO4FbrjkmEps0?m=slm-pNLsWVV7ue9mSprIxmgQ
pHBS1722 CtaggerV6-NPM1-mTagRFP	This study	https://benchling.com/s/seq-YB4p228xrZwmbmzaspC5?m=slm-95nXWoEW7JuAg1fjnx1E
UBTF N-sfGFP donor	Leonetti Lab	https://benchling.com/s/seq-LPCVx6eP5X60vB8V48Z4
FBL C-Halo donor	Leonetti Lab	https://benchling.com/s/seq-fr1086bTDcEuTHiGmYpy
NPM1 C-mTagRFP donor	Leonetti Lab	https://benchling.com/s/seq-tkR3x4eaRuAhf6FJ5jUU
pHBS1710 SpCas9-sgFBL-HDR	This study	https://benchling.com/s/seq-OZQjgEzeSSyzAu0dW70A?m=slm-iuolZPftKdnnCZCay8mA
pHBS1711 SpCas9-sgNPM1	This study	https://benchling.com/s/seq-ZuYQK3HxvpywE3vOy8Xb?m=slm-6z346KIatYtEadthTb8h
GFP	Glaunsinger Lab	https://benchling.com/s/seq-PrhlQjix21Occ3gLKuIs?m=slm-qsZV2rTUXpu2HqJg1lLj
GFP-FBL	Addgene	26,673
GFP-NPM1	Addgene	17,578
Software and algorithms		
cellSens	Olympus	Version 3.2
LAS X	Leica	Version 4.13
Mathematica	https://wolfram.com	Version 12.2
MATLAB	https://mathworks.com	Version 9.3
Python	https://python.org	Version 3.8.5
Numpy	https://numpy.org	Version 1.21.2
Pandas	https://pandas.pydata.org	Version 1.3.4
MatPlotLib	https://matplotlib.org	Version 3.3.1
SciPy	https://scipy.org	Version1.7.1
SciKit Image	https://scikit-image.org	Version 0.17.2
JupyterLab	https://jupyter.org	Version 3.2.1
Custom code	This study	https://github.com/RohatgiLab/2022_Schmidt_Oxaliplatin
Other		
PRISM drug screen dataset	https://depmap.org	Version 19Q4
CCLE expression dataset	https://depmap.org	Version 21Q4

## Highlights

- Oxaliplatin disrupts the multi-phase organization of nucleoli
- Oxaliplatin indirectly inhibits rRNA synthesis
- Oxaliplatin modifies the nucleolar scaffold protein FBL and impairs its phase separation
- FBL overexpression alleviates oxaliplatin toxicity
