# Supplementary material for: Oxaliplatin disrupts nucleolar function through biophysical disintegration
Source: Cell Rep. Author manuscript; Available in PMC 2022 Dec 14. (PMC9749789; doi:10.1016/j.celrep.2022.111629)
Supplement: MMC1 [file NIHMS1848913-supplement-MMC1.pdf]

Cell Reports, Volume *41*

Supplemental information

Oxaliplatin disrupts nucleolar function through biophysical disintegration

H. Broder Schmidt, Zane A. Jaafar, B. Erik Wulff, Jason J. Rodencal, Kibeom Hong, Mohammad O. Aziz-Zanjani, Peter K. Jackson, Manuel D. Leonetti, Scott J. Dixon, Rajat Rohatgi, and Onn Brandman

Supplemental Figures

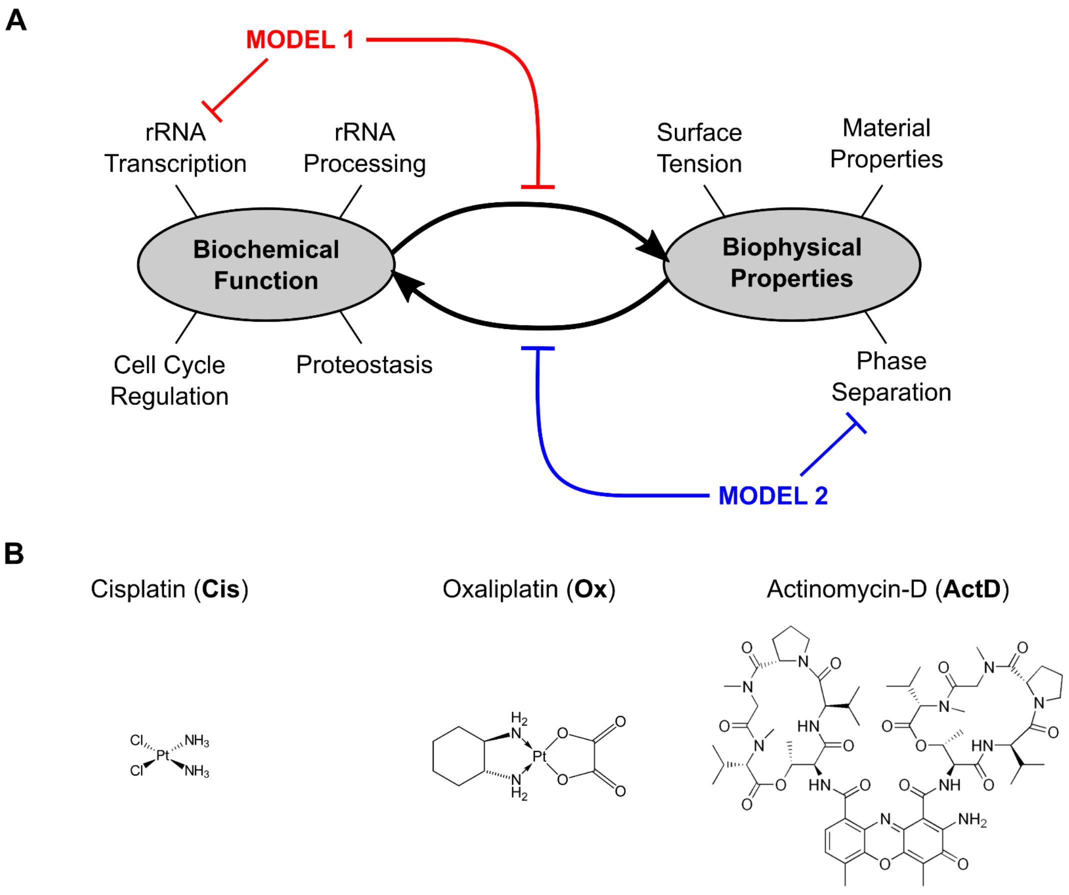

Figure S1. *Models for the oxaliplatin mechanism of action; related to Figures 1-7.*

(**A**) Schematic showing the two models that we propose to explain how oxaliplatin may act as a transcription inhibitor at the level of nucleoli. In model 1, oxaliplatin acts similarly as actinomycin D and directly shuts-down rRNA transcription, in turn disrupting nucleolar biophysics because rRNA is a key determinant of nucleolar form. In model 2, oxaliplatin interferes with nucleolar biophysics, for example by interfering with nucleolar phase separation, thereby indirectly inhibiting rRNA transcription.

(**B**) Chemical structures of the small molecule drugs used in this study.

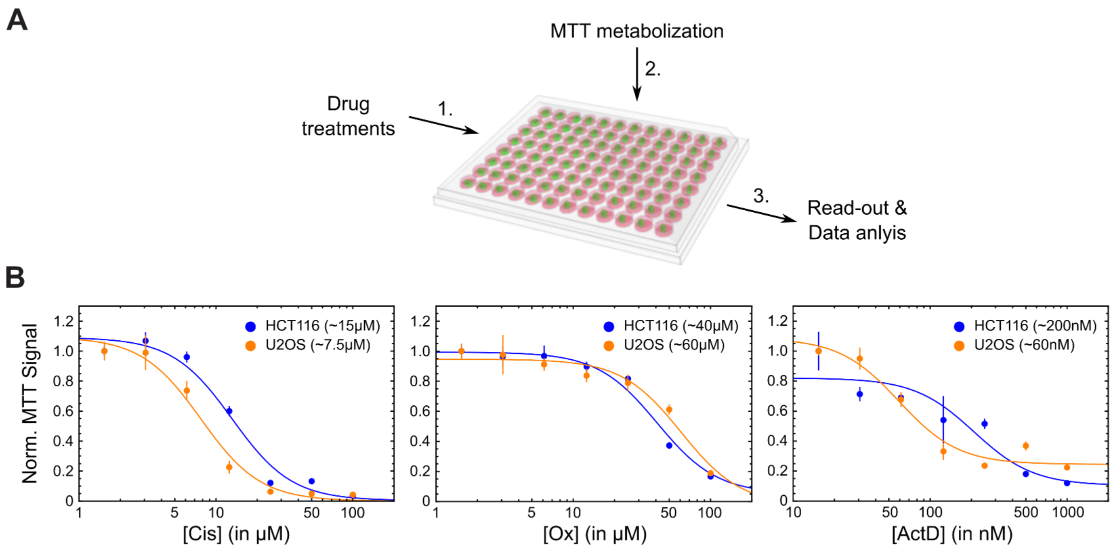

Figure S2. *Sensitivity of HCT116 and U2OS cells to cisplatin, oxaliplatin and actinomycin D; related to Figures 1-7.*

(**A**) Workflow summary of MTT assay to measure cell viability after drug treatments.

(**B**) Dose-response curves for HCT116 and U2OS cells treated with the indicated types and amounts of drugs for 48 hours. Data points represent the mean values from four replicates, error bars the standard deviation and curves a nonlinear dose-response fit to the data (see Methods). Numbers indicate the EC50 values derived from the fits.

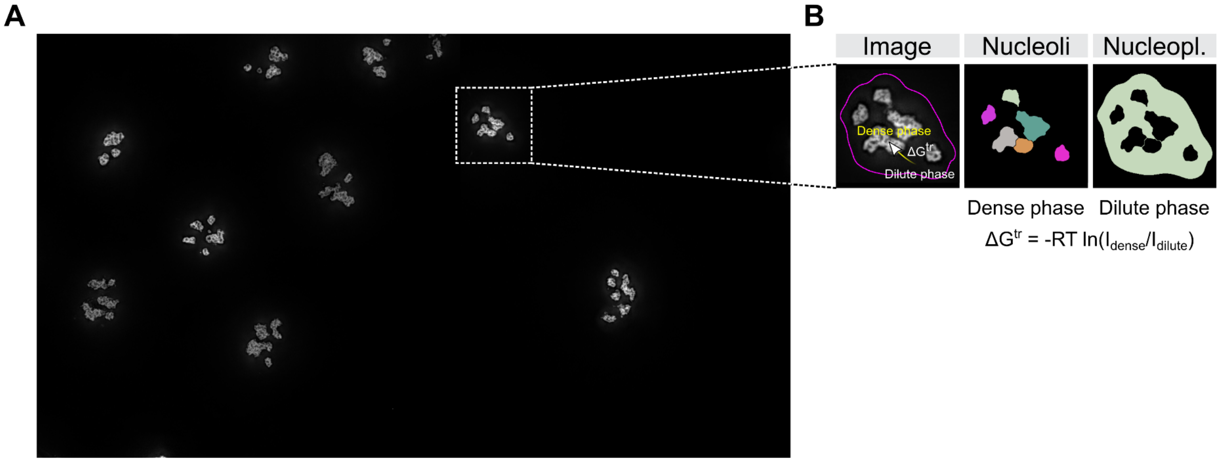

Figure S3. *Image segmentation and quantification approach; related to Figures 1-5.*

(**A**) Collage of deconvoluted images showing nucleoli in untreated U2OS cells, as visualized via NPM1-RFP (tagged at its endogenous locus).

(**B**) Schematic outlining the segmentation approach for measuring the nucleolar (dense phase) and nucleoplasmic (dilute phase) fluorescence intensity (I) signals to estimate the transfer free energy ΔG^{tr} using the molar gas constant (R) and temperature (T).

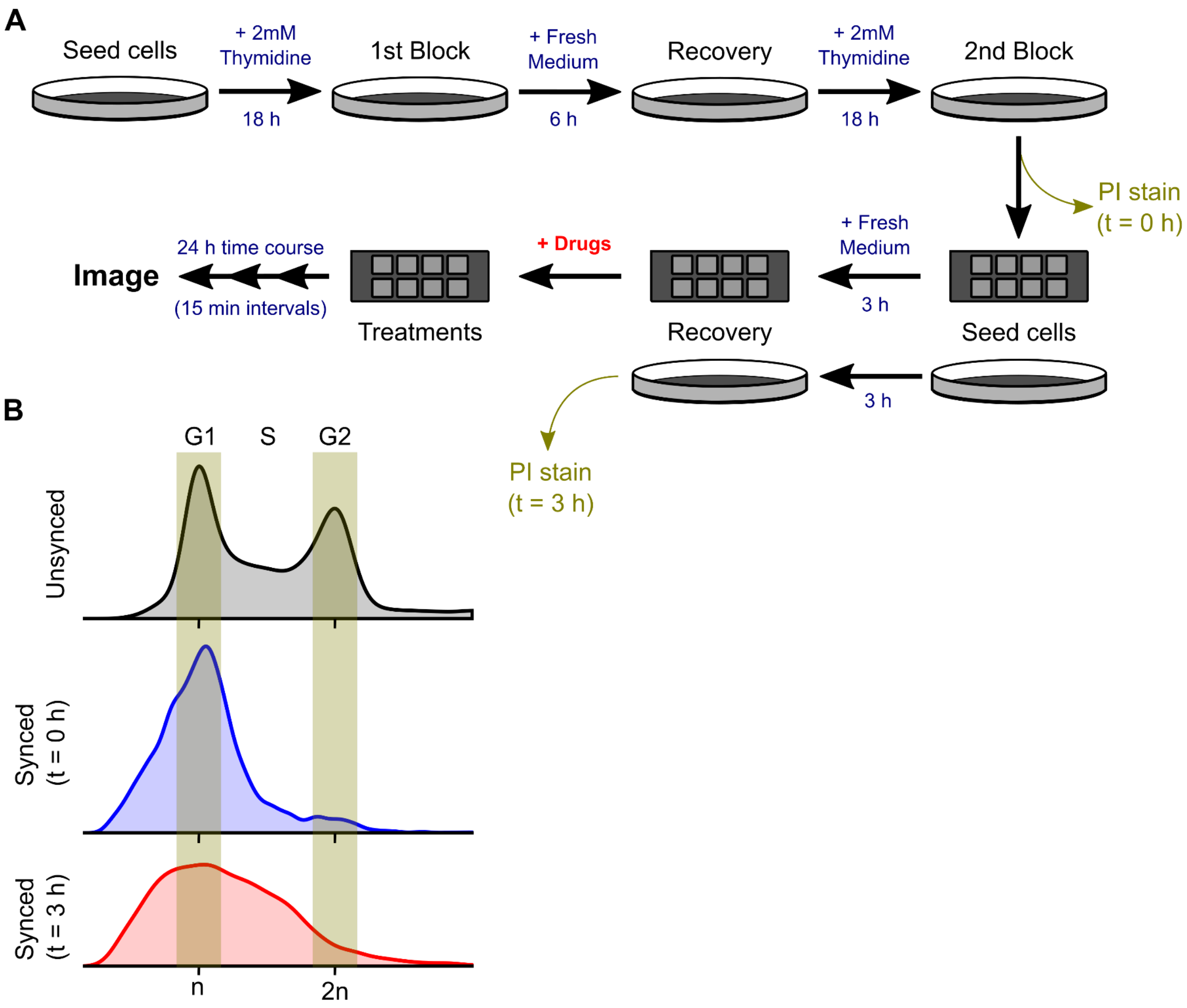

Figure S4. *Synchronization of U2OS cells for analysis of nucleolar dynamics during the cell cycle; related to Figure 3.*

(**A**) Flow chart outlining the synchronization process using the double-thymidine block method. After the second block, cells were seeded for analysis by live cell imaging and flow cytometry.

(**B**) Histograms showing the DNA content of unsynchronized and synchronized cells stained with propidium iodide and analyzed with flow cytometry as outlined in (A).

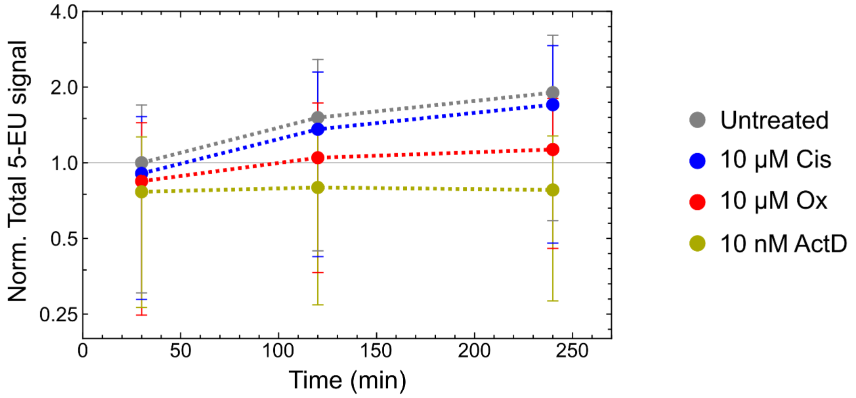

Figure S5. *Changes in total nucleolar transcription over time; related to Figure 5.*

Change of total 5-EU signals per cell over time in U2OS cells treated with the indicated drugs. 5-EU data is normalized to the earliest measurement (30 min) in untreated cells. Plot markers represent median values and error bars median deviation. At least 771 nucleoli per treatment and time point were quantified. See Supplemental File 1 for statistics.

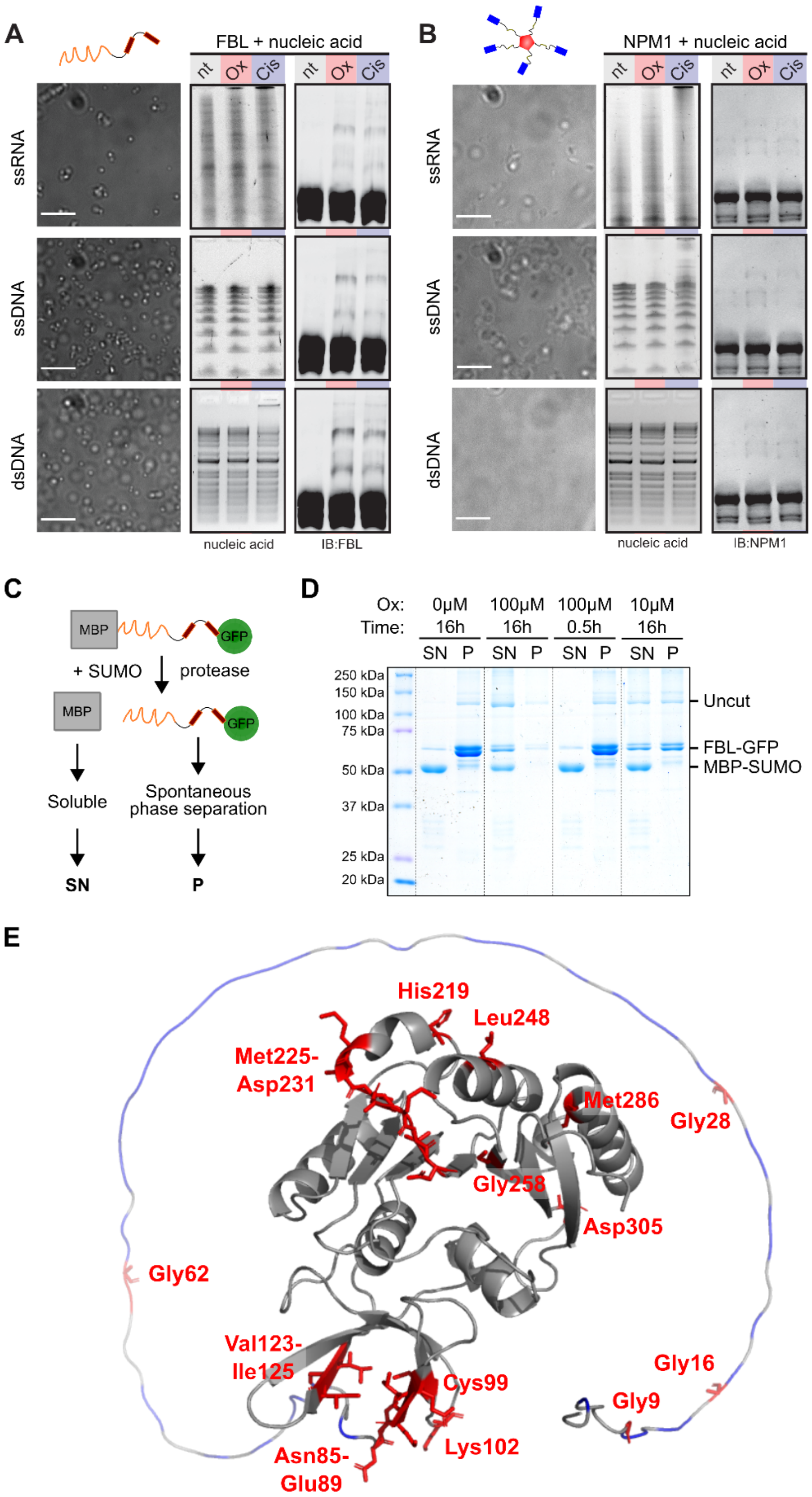

Figure S6. *Biochemical analysis of nucleic acid and protein modification by oxaliplatin and cisplatin; related to Figure 6.*

(**A**) and (**B**) DIC imaging, Hepes/triethanolamine-formaldehyde AGE (ssRNA), urea-AGE (ssDNA), standard AGE (dsDNA), and SDS-PAGE (protein) to assess platination and phase separation of FBL (A) and NPM1 (B) in the presence of the indicated nucleic acids after treatment with oxaliplatin and cisplatin. Nucleic acids were stained with SYBR Green and proteins detected by immunoblotting. Scale bars: 5 µm.

(**C**) Schematic of FBL *in vitro* phase separation assay. Recombinant GFP-tagged, full-length FBL was initially purified from *E. coli* containing an N-terminal maltose binding protein (MBP) tag. To trigger phase separation, the MBP tag was cleaved off by addition of SUMO protease.

(**D**) Phase separation of FBL-GFP (at 10 μM) after modification with oxaliplatin at the indicated conditions, analyzed by SDS-PAGE and Coomassie staining after centrifugation at 20,000 × *g* for 30 min.

(**E**) Mapping of oxaliplatin modifications onto the AlphaFold2 prediction of FBL structure (P22087). FBL in gray, experimentally verified sites modified by oxaliplatin in red, glycine residues in the intrinsically disordered region in blue.

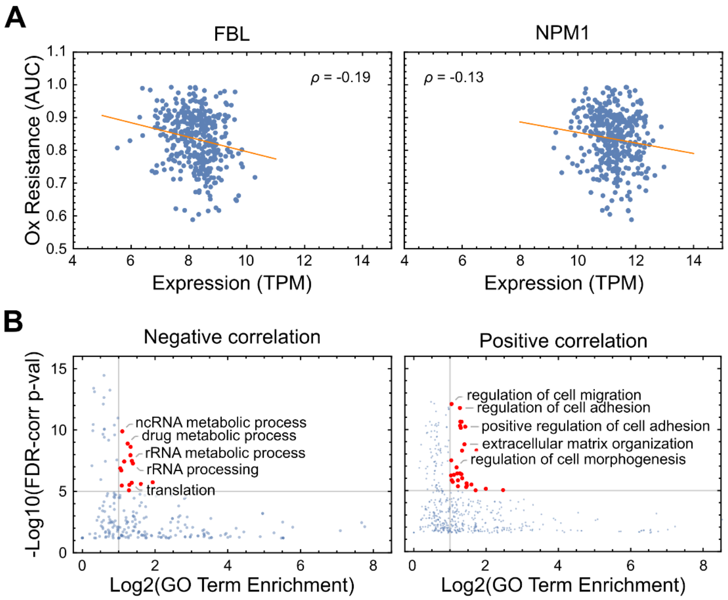

Figure S7. *Correlation between transcript levels and oxaliplatin resistance across cancer cell lines; related to Figure 7.*

(**A**) For 371 cancer cell lines in the DepMap repository (depmap.org/portal), the expression levels of 19,177 transcripts (in TPM) were correlated with oxaliplatin resistance (quantified by the area under the dose-viability-curve, AUC; data from the PRISM high-throughput drug screen), as exemplified here for the transcripts encoding the nucleolar marker proteins FBL and NPM1. Data points denote cancer cell lines, orange lines a linear fit to the data points, and ρ the Pearson correlation coefficient.

(**B**) Enrichment (x-axis) and FDR-corrected p-value (y-axis) of the GO terms associated with transcripts that are negatively or positively correlated with oxaliplatin resistance (based on ρ). Data points represent GO terms, with the most significant cellular processes highlighted in red.
